# Impacts of heat, drought, and combined heat–drought stress on yield, phenotypic traits, and gluten protein traits: capturing stability of spring wheat in excessive environments

**DOI:** 10.3389/fpls.2023.1179701

**Published:** 2023-05-19

**Authors:** Sbatie Lama, Fernanda Leiva, Pernilla Vallenback, Aakash Chawade, Ramune Kuktaite

**Affiliations:** ^1^ Department of Plant Breeding, Swedish University of Agricultural Sciences, Lomma, Sweden; ^2^ Lantmännen Lantbruk, Svalöv, Sweden

**Keywords:** wheat, phenotyping, gluten protein quality, heat, drought, heat-drought

## Abstract

Wheat production and end-use quality are severely threatened by drought and heat stresses. This study evaluated stress impacts on phenotypic and gluten protein characteristics of eight spring wheat genotypes (Diskett, Happy, Bumble, SW1, SW2, SW3, SW4, and SW5) grown to maturity under controlled conditions (Biotron) using RGB imaging and size-exclusion high-performance liquid chromatography (SE-HPLC). Among the stress treatments compared, combined heat–drought stress had the most severe negative impacts on biomass (real and digital), grain yield, and thousand kernel weight. Conversely, it had a positive effect on most gluten parameters evaluated by SE-HPLC and resulted in a positive correlation between spike traits and gluten strength, expressed as unextractable gluten polymer (%UPP) and large monomeric protein (%LUMP). The best performing genotypes in terms of stability were Happy, Diskett, SW1, and SW2, which should be further explored as attractive breeding material for developing climate-resistant genotypes with improved bread-making quality. RGB imaging in combination with gluten protein screening by SE-HPLC could thus be a valuable approach for identifying climate stress–tolerant wheat genotypes.

## 1 Introduction

Wheat (*Triticum aestivum* L.) is the third most common cereal produced worldwide, with more than 771 million tonnes harvested in 2021 (https://www.fao.org/faostat). Wheat provides approximately 20% of global total human dietary calories and 21% of daily protein consumption (Shiferaw et al., 2013). With increasing population and urbanization, consumption and associated demand for wheat-based food products are increasing (Peña, 2007); thus, sustaining wheat production and quality is important for ensuring food security. With ongoing climate change and global warming, extreme climate events and abiotic stresses are becoming more severe and unpredictable (Le Gouis et al., 2020). Climate events such as heat, drought, excessive rainfall, and high atmospheric concentrations of CO_2_ are already affecting the production and quality of wheat worldwide (Yadav et al., 2020). The extent of the losses depends on the plant growth stage affected and the severity of the stress (Wahid et al., 2007; Lan et al., 2022). Therefore, the development of wheat genotypes that are resistant to various abiotic stresses is crucial for food security under ongoing climate change.

Among the abiotic stresses imposed by climate change, heat and drought stresses are considered to cause the most damage to wheat growth and development (Mamrutha et al., 2020). Drought during stem elongation and heat stress during the grain-filling stage have been identified as particularly important environmental factors affecting the yield and quality of wheat (Guzmán et al., 2016; Le Gouis et al., 2020). This is because drought and heat impair the growth and development of different wheat plant organs, the rate of photosynthesis, fertility, the number of spikes, grain-filling, and nutrient uptake by the plant (Hurkman and Wood, 2011; Guzmán et al., 2016; Lan et al., 2022). Yield losses due to individual or combined heat–drought stresses have been observed in multiple countries in Europe, including Finland, Sweden, France, Belgium, and Switzerland (Kumar et al., 2020; Le Gouis et al., 2020; Lama et al., 2022). Areas such as the Mediterranean and southern Europe are experiencing higher impacts of heat–drought stress than other regions, causing major economic and food production losses [EEA (European Environment Agency), 2019]. A 1°C–3°C rise in mean global air temperature is suggested to decrease wheat production by up to 28% (Shew et al., 2020; Zhang et al., 2022). In fact, in a previous study by our research group on field-grown wheat in Sweden, a yield reduction of up to 40% was observed under combined heat–drought conditions compared with rainy and cold conditions (Lama et al., 2022).

Wheat yield is generally the main focus in research due to its direct relationship to food security (Asseng et al., 2019). However, manufacturers of different wheat-based food products, such as bread and pasta, require wheat flours with a specific protein quality (Johansson et al., 2020). Wheat quality is mainly determined by its major protein, gluten, the quantity and quality of which are often negatively impacted by heat and drought. For instance, the relatively high content of protein and strong gluten required in wheat bread flour (Kuktaite et al., 2004) is severely affected by intense heat and drought stresses (Lama et al., 2022). Total protein content and gluten content are reported to increase by 65% and 32%, respectively, under combined heat–drought stress compared with control conditions (Sattar et al., 2020). In greenhouse studies, the relative proportions of different types of proteins, such as high molecular weight (HMW) and low molecular weight (LMW) glutenins, and omega-, alfa/beta- and gamma-gliadins, have been found to increase under heat stress (35°C day temperature) compared with a control environment (Zhao et al., 2022).

The susceptibility of wheat plants to abiotic stresses depends mainly on the duration, frequency, and intensity of the stress conditions to which the plants are exposed (Barnabás et al., 2008; Farooq et al., 2009; Qaseem et al., 2019). Due to the adaptive metabolic and physiological mechanisms that wheat plants have developed, physiological responses at different developmental stages can differ between genotypes (Rampino et al., 2006). Plants under drought stress decrease their leaf area and increase canopy temperature in order to prevent water loss (Anjum et al., 2011), although heat stress combined with appropriate irrigation can increase transpiration rates and decrease canopy temperature (Singh et al., 2007). In combined heat–drought stress, there is an extreme effect on the physiological responses of wheat plants at all growth and reproductive stages (Rizhsky et al., 2002; Prasad et al., 2011). In combination, these two stresses can have complex contradictory effects compared with when they occur separately (Zhou et al., 2017).

Physiological traits of wheat, such as growth and characteristics related to yield, are commonly assessed by visual or manual annotation methods, but these tend to be subjective, time-consuming, and laborious (Dhondt et al., 2013). Therefore non-destructive remote and proximal phenotyping techniques are becoming more popular and more widely used (Armoniené et al., 2018; Leiva et al., 2021; Tao et al., 2022). Such methods have been developed to extract data mainly from images in the visual and electromagnetic spectrum on several plant traits with high accuracy, reliability, and time resolution (Humplík et al., 2015; Chawade et al., 2019; Reynolds et al., 2019). Cameras that provide spectral information for each pixel in an image, such as multispectral and hyperspectral data, have proven to be a valuable tool for studying plants under abiotic stresses (Cao et al., 2019). The main difference between multispectral and hyperspectral data is the number of bands in the light spectrum (5–10 bands and hundreds, respectively) (Sara et al., 2021). However, these cameras are expensive and require sophisticated statistical methods for data processing (Zubler and Yoon, 2020). Low-cost digital red, green and blue (RGB) cameras can readily estimate plant shoot biomass, development, and growth rate and can be a suitable tool for mapping plant responses under heat and drought (Blum et al., 1997; Humplík et al., 2015).

Phenotypic traits such as plant growth and yield and grain quality characteristics are important for sustaining the supply of wheat-based food products. Thus, a clear understanding of wheat response mechanisms to heat, drought, and combined heat–drought stresses is essential. In addition, since phenotypic traits (e.g., yield/grain weight) and wheat protein quality parameters are usually negatively related (Daniel and Triboi, 2000; Johansson et al., 2005), the stability of these attributes under climate change is complex and needs to be investigated.

The aim of this study was to determine the effects of individual and combined heat–drought stresses on phenotypic plant growth characteristics, yield, and gluten protein quality parameters of spring wheat genotypes grown in highly controlled environments. Plant growth and development under heat, drought, and combined heat–drought stresses were monitored using RGB imaging, and the gluten protein quality characteristics of wheat grain were assessed by size-exclusion high-performance liquid chromatography (SE-HPLC). The stability of yield and of gluten protein quality parameters for different spring wheat genotypes grown under stress conditions was also evaluated.

## 2 Materials and methods

### 2.1 Plant material

Eight spring wheat genotypes (Diskett, Happy, Bumble, SW1, SW2, SW3, SW4, and SW5) developed in the breeding program at Lantmännen Lantbruk, Svalöv, Sweden, were evaluated. The selected genotypes represented a range in gluten strength, as identified when grown in the field in our previous study (Lama et al., 2022). Diskett, Bumble, and SW3 represented genotypes with unstable gluten strength (>5% variation between years), while Happy, SW1, SW2, SW4, and SW5 represented genotypes with stable gluten strength (<5% variation between years) (Lama et al., 2022).

### 2.2 Experimental design and description of stress environments

The spring wheat plants were grown in a randomized complete block design under three stress environments (heat, drought, and combined heat–drought), which were applied simultaneously. The plants were grown in plastic pots (20 cm × 16 cm, volume 3.5 L) in peat-based soil, with three plants per pot. Two weeks after emergence, a plant cone (61 × 25 cm) was inserted in each pot to support the growing plants. Each pot was considered a biological replicate, and four biological replicates were used per genotype.

**Figure 1:**
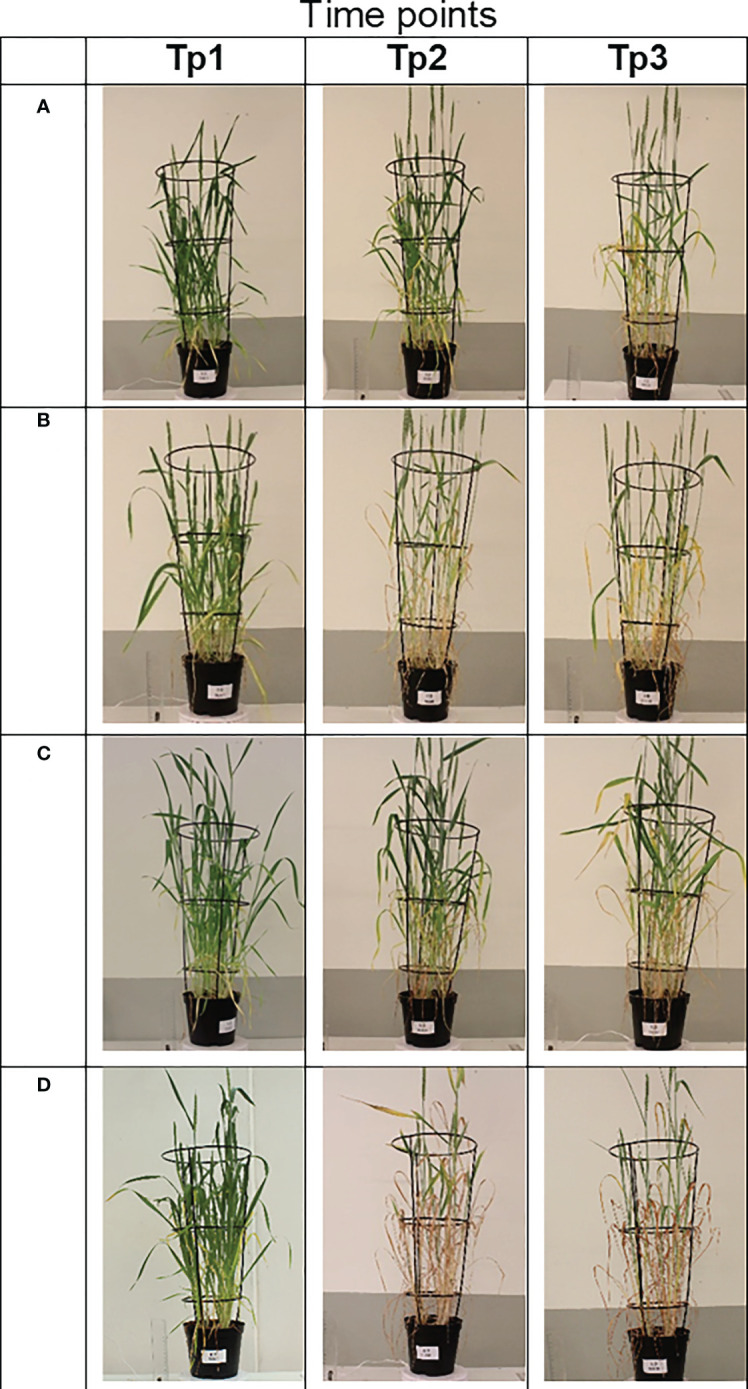
Digital red, green, and blue (RGB) images taken at time points Tp1–Tp3 (Tp1 = no stress, Tp2 = after 5 days of stress treatment, and Tp3 = after 8 days of recovery) of the spring wheat genotype Diskett in the heading stage (Zadoks 50) under different growing conditions (stress treatments). **(A)** control, **(B)** drought, **(C)** heat, and **(D)** combined heat–drought stress.

The pots containing the eight genotypes were grown in two Biotron climate chambers with artificial lighting from February to June 2020 at Swedish University of Agricultural Sciences (SLU), Alnarp, Sweden (**Tables S1, S2**; **Supplementary Information** (SI)). Growing conditions in terms of temperature, humidity, and day length (hours) were based on mean 5-year (2016–2020) weather data for the growing period in Malmö, Sweden (22 April–11 August) obtained from the Swedish Meteorological and Hydrological Institute (SMHI) (www.smhi.se). The daylight intensity of 400 μmol m^-2^s^-1^, produced with light-emitting diode (LED) lights, was provided during the growing period. Until the start of the stress treatments, all plants were watered every 2 days with approximately 500 ml water per pot. The drought, heat, and combined heat–drought stress treatments (information below) were introduced at the beginning of heading stage (Zadoks 50) (Zadoks et al., 1974), at approximately 56 days after sowing, and were applied for 5 days, resulting in signs of stress in the plants (dry, yellow leaves) (**Figure 1A**).

#### 2.2.1 Heat

In this treatment, the temperature was kept at 29°C during day and night for 5 days and the 56-day-old plants were watered as in the control (500 ml water/pot every 2 days) (**Table S1**). After 5 days, the temperature was returned to the control level (15°C) (**Table S1**).

#### 2.2.2 Drought

Plants assigned to the drought treatment began heading (Zadoks 50) slightly earlier than the plants in the other treatments. Drought stress was thus applied to 51-day-old plants, by stopping all watering of the plants for 5 days. After 5 days, normal watering was resumed (500 ml water/pot every 2 days).

#### 2.2.3 Combined heat–drought stress

In this treatment, the 56-day-old plants received no water for 5 days and the temperature was maintained at 29°C during day and night (**Table S1**). After 5 days, normal watering was resumed and the temperature was set to control conditions (**Tables S1, S2**).

Wheat plants still growing 8 days after stopping the stress treatments were considered recovered plants. The digital biomass of all plants was recorded at three time points (Tp1 = no stress, Tp2 = after 5 days of stress treatment, and Tp3 = after 8 days of recovery) (**Table 1**).

**Table 1:** Plant growth stage (Zadoks scale) and age of plants (days) in the different treatments at the three time points (Tp1–Tp3) when the digital biomass of spring wheat plants was measured.

Time point (plant status)	Zadoks wheat growth stage	Combined heat–drought	Treatment Heat	Drought
Tp1 (non-stressed)	Heading (Zadoks 50)	56	56	51
Tp2 (stressed)	Heading (Zadoks 59)	61	61	56
Tp3 (recovered)	Grain development (Zadoks 70)	70	70	65

### 2.3 Image acquisition

The biomass of the wheat plants was assessed digitally from the top and side through RGB imaging in a laboratory with LED light, using two Canon EOS 1300D digital single-lens reflex cameras with an 18–55 mm kit lens (Armoniené et al., 2018). The cameras were mounted on a SpaceArm (Tristar) at 1 m for the top view and on a tripod at 1.5 m for the side view. Plant pots were placed manually on a top-quality Intelligent 360 Photography turntable platform (Shenzhen Comxim Technology Co., Ltd., Shenzhen, Guangdong, China) and individually photographed using the DigiCamControl software (Istvan, 2014). During the side-view imaging, the plant pot was rotated by 90° four times, to acquire four images (front, right, left, and back projections). Shadows and light differences were adjusted by camera settings and exposure. For both cameras, focal length was set at 18 mm and ISO 1600, while light exposure was set as F-Stop f/13 and exposure time 1/60 s for the top-view camera, and F-Stop f/8 and exposure time 1/40 for the side-view camera. The images obtained were stored in JPEG format, using resolution 3,456 × 2,304 pixels for top projection and 5,184 × 3,456 pixels for side projection.

### 2.4 Image processing

The digital biomass of each plant was automatically extracted with EasyLeaf software (Easlon and Bloom, 2014). Since all images were acquired under the same light conditions, the red and green thresholds in the software and the individual ratios of RGB values [green/red (G/R) and green/blue (G/B)] were set using the first image of each measuring occasion (Tp) and then processed in batch. Finally, projected leaf area (PLA) was obtained from the average of the five plant images (top at 0° and four sides at 90°) as


$$PLA=\sum_{1}^{n}pla$$

### 2.5 Phenotypic traits

The height of all three plants in each pot was measured with a ruler as the distance from the soil surface to the tip of the spike, excluding the awns. To measure the spike length (mm), the tallest spike of each plant in the pot was selected and the length of the spikes was recorded from the base of the rachis to the tip of the terminal spikelet, excluding the awns. Spike width (mm) was measured on these same spikes, at a point halfway along the spike height. The weight of fresh biomass (g), including the weight of spikes (g), per pot was recorded. Thousand kernel weight (TKW) (g) was calculated as described by Wu et al. (2018). The number of spikes was counted for each plant per pot, and grain yield (g) was recorded per plant.

### 2.6 Gluten protein parameters in the flour

SE-HPLC was used to evaluate the gluten protein characteristics of the harvested grain. Seeds from the different genotypes grown at different stresses and control samples were milled into flour using a homogenizer (Mixer Mill MM 400, Retsch) for 30 s at 30 Hz. The flours were freeze-dried (Cool safe Pro, LaboGene) for 24 h in order to remove all moisture prior to SE-HPLC analysis. A two-step gluten protein extraction method was performed according to Lama et al. (2022), with some modifications where collected supernatant (after first and second steps) in SE-HPLC vials was heated at 80°C for 2 min (to inactivate proteases) in a water bath according to Islas-Rubio et al. (2006). Samples were run on the SE-HPLC system in triplicate. The concentrations of total SDS-extractable protein (TOTE), total SDS-unextractable protein (TOTU), %UPP (the percentage of SDS-unextracted polymeric proteins in total polymeric proteins), and %LUMP (the percentage of large SDS-unextracted large monomeric proteins in total large monomeric proteins) were calculated according to Lama et al. (2022). Total polymeric proteins (TPPs) and total monomeric proteins (TMPs) were calculated as LPP + SPP + LPPs + SPPs and LMP + SMP + LMPs + SMPs, respectively, where LPP, SPP, LMP, and SMP are SDS-extractable large polymeric proteins, small polymeric proteins, large monomeric proteins, and small monomeric proteins, respectively, and LPPs, SPPs, LMPs, and SMPs are the corresponding SDS-unextractable form.

### 2.7 Statistical analysis

Statistical analyses were performed using the software R (Team, 2013). Principal component analysis (PCA) using the R packages FactoMineR and a two-way analysis of variance was conducted, with Tukey’s *post hoc* test (p< 0.05), to assess the effect of different treatments on the gluten protein parameters and phenotypic traits. Spearman correlation analysis (p< 0.05) (R package Corrplot) was applied for all gluten protein parameters and phenotypic traits in plants in each treatment. Genotype main effect plus genotype by environment interaction (GGE) biplots analysis (R package Metan) was performed to evaluate the stability of the studied genotypes in the different growing environments. The selected GGE tools were “mean vs. stability,” “which-won-where view of the GGE biplots,” and “ranking genotypes.” These tools facilitate the identification of the optimal genotypes based on performance and stability, identify the optimal genotype for each growing environment, and rank them according to suitability for a growing environment.

## 3 Results

### 3.1 Digital biomass assessment

The digital wheat biomass assessed by RGB imaging at time points Tp1, Tp2, and Tp3 differed between the genotypes for the different stress conditions tested (**Figure 1**). The greatest differences in plant appearance were observed for the genotype Diskett in the combined heat–drought stress treatment (**Figure 1**; at Tp2 and Tp3). The impact of drought was similar to that of combined heat–drought at Tp2 and Tp3, with both treatments resulting in semi-dry plants (**Figures 1B, D**). The heat stress treatment had mild effects on the plants (**Figure 1C**).

**Figure 2:**
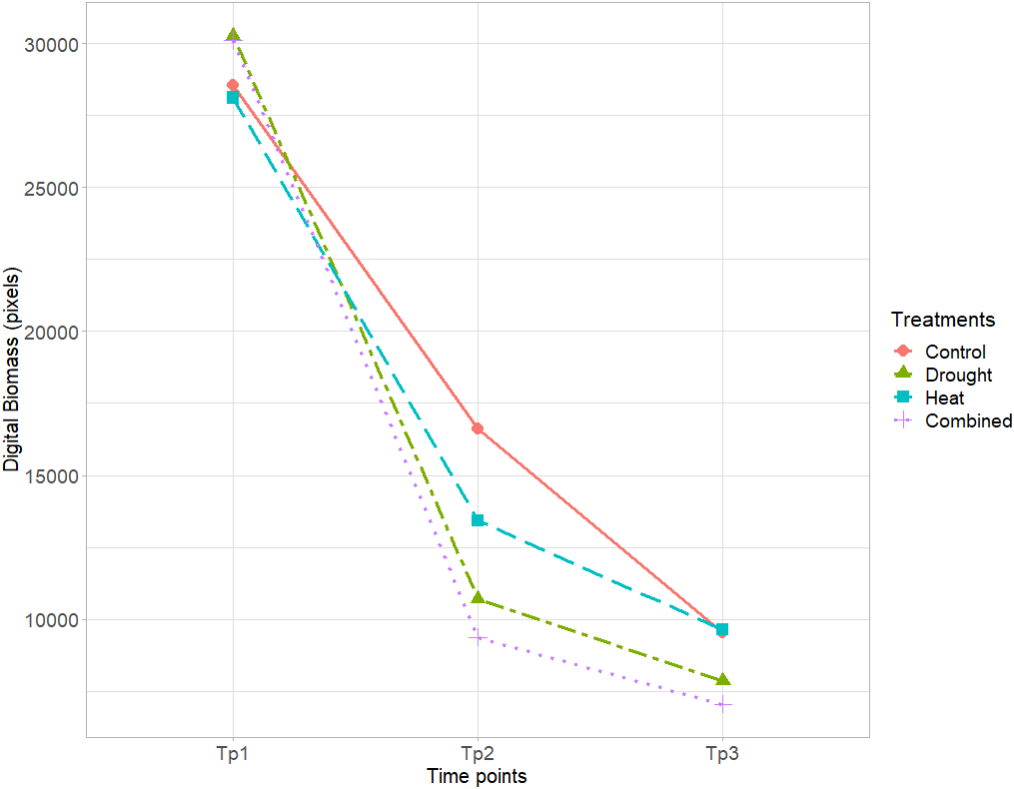
Mean digital biomass (pixels) of the eight wheat genotypes in the control, drought, heat, and combined heat–drought stress treatments, based on RGB imaging at time points Tp1–Tp3 (Tp1 = no stress, Tp2 = after 5 days of stress treatment, and Tp3 = after 8 days of recovery).

Mean digital biomass measured at flowering (anthesis) was similar for all genotypes at Tp1 and decreased for all genotypes at both Tp2 and Tp3. The greatest impact of the stress treatments was observed in the drought and combined heat–drought treatments, followed by heat (**Figure 2**). The greatest variation in mean digital biomass between the different genotypes was observed under drought and combined heat–drought (**Figure 2**).

**Figure 3:**
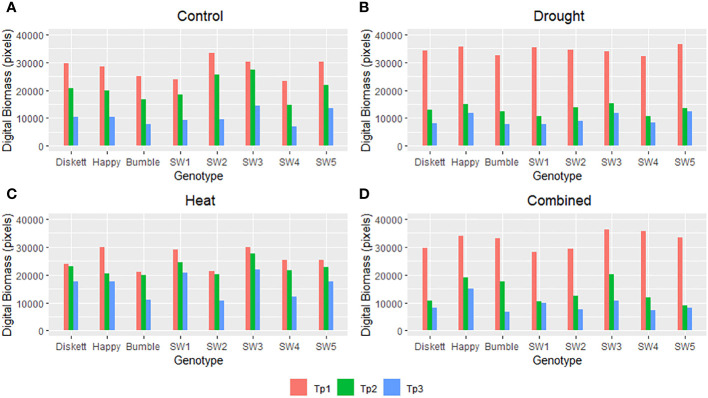
Digital biomass (pixels) of the eight spring wheat genotypes in the **(A)** control, **(B)** drought, **(C)** heat, and **(D)** combined heat–stress treatments, based on RGB imaging at time points Tp1–Tp3 (Tp1 = no stress, Tp2 = after 5 days of stress treatment, and Tp3 = after 8 days of recovery).

Under control conditions, rather similar digital biomasses at Tp1–Tp3 were observed for all genotypes except SW1 and SW3 (**Figure 3A**). Genotype SW3 had relatively higher digital biomass at Tp2 and Tp3, while in SW1, it was somewhat lower. Drought reduced the digital biomass of all genotypes compared with the control and especially that of SW1 and SW4 (**Figure 3B**). Heat stress had almost no impact at Tp1 and Tp2 but some impact at Tp3, especially for Bumble, SW2, and SW4 (**Figure 3C**). Diskett, SW1, and SW3 showed relatively similar digital biomass at Tp1–Tp3 in the heat stress treatment (**Figure 3C**). In the heat–drought treatment, the digital biomass was significantly reduced in all genotypes at Tp2 and Tp3, although the reduction observed between Tp2 and Tp3 was somewhat smaller for Happy (**Figure 3D**).

### 3.2 Effect of genotypes and stress conditions on gluten protein parameters

The impact of genotype (G) and stress environment (E) on the protein parameters was significantly dominated by the individual effect and not by G and E interaction (G × E) (**Tables 2**, **S3** in SI). A significant impact of G × E interaction was observed on the monomer-to-polymer (Mon/pol) ratio (p< 0.01) and TOTU (p< 0.05) (**Table 2**). The G × E interaction had a significant impact in particular on unextractable gluten polymers (LPPs and SPPs) and SMPs (**Table S3**).

**Table 2:** Analysis of variance (ANOVA) results for the effect of genotype (G), treatment (E), and their interaction (G × E) on gluten protein parameters [total amount of SDS-extractable (TOTE) and SDS-unextractable (TOTU) protein, total polymeric protein (TPP), total monomeric protein (TMP), SDS-unextractable polymeric protein (%UPP), large SDS-unextractable polymeric protein (%LUPP), large SDS-unextractable monomeric protein (%LUMP), and monomer-to-polymer ratio (Mon/pol)] of wheat grown under control, heat, drought, and heat–drought stress conditions.

Factor	Df	TOTE 10^16^	TOTU 10^15^	TPP 10^15^	TMP 10^15^	%UPP 10^3^	%LUPP 10^3^	%LUMP 10^2^	Mon/ pol
Genotype (G)	7	0.53***	0.60***	0.98***	3.05***	1.65***	3.23***	0.80***	0.38***
Treatment (E)	3	1.33***	2.49***	3.80***	10.62***	1.88***	3.87***	0.22**	0.38***
G × E	21	0.26	0.76*	0.95	1.87	0.84	0.87	0.36	0.72**
Residuals	96	1.09	1.78	2.77	7.41	2.45	3.60	1.44	1.28

***, **, and * indicate significance at p< 0.001, p< 0.01, and p< 0.05, respectively. Df, degrees of freedom.

All parameters were measured by size-exclusion high-performance liquid chromatography (SE-HPLC).

The evaluation of the effect of the different treatments on the gluten protein parameters by Tukey’s *post hoc* test indicated a major impact of the combined heat–drought stress conditions on the gluten protein parameters, with significant effects on TOTE, TOTU, TPP, TMP, and %UPP in comparison with the other environmental conditions (**Table 3**).

**Table 3:** Results of Tukey’s *post hoc* tests of different stress environments (drought, heat and combined heat–drought) on gluten protein parameters (TOTE and TOTU protein, TPP, TMP, %UPP, %LUPP, %LUMP, and Mon/pol) in wheat (evaluated by SE-HPLC).

Factor	TOTE 10^7^	TOTU 10^7^	TPP 10^7^	TMP 10^7^	%UPP 10^3^	%LUPP 10^3^	%LUMP 10^2^	Mon/pol
Control	6.15 b	1.22 c	2.44 b	4.92 bc	31.06 c	34.84 c	9.45 b	2.02 a
Drought	5.81 b	1.28 bc	2.32 b	4.75 c	35.62 b	41.18 b	9.50 b	2.06 a
Heat	6.49 b	1.54 b	2.62 b	5.41 b	37.93 b	46.07 a	10.23 ab	2.08 a
Combined	8.43 a	2.32 a	3.70 a	7.06 a	41.65 a	49.46 a	10.36 a	1.93 b

Different letters indicate significant difference at p< 0.05.

A similar impact of heat–drought stress was observed on most gluten parameters studied (**Table 3**) and some impact of heat stress on TMP, %LUPP, SPP, and LMPs (**Tables 3**, **S4** in SI) in comparison with drought stress. Surprisingly, no significant differences between the drought stress treatment and the control were found for any of the gluten protein parameters studied except %UPP and %LUPP (**Table 3**). Lower amounts of %UPP and %LUPP (gluten strength) and, somewhat unexpectedly, a higher amount of LPP was found under the control environment compared with the drought and heat stress treatments (**Tables 3**, **S4**).

### 3.3 Relationship between genotypes, phenotypic traits, and gluten parameters under different stress environments

**Figure 4:**
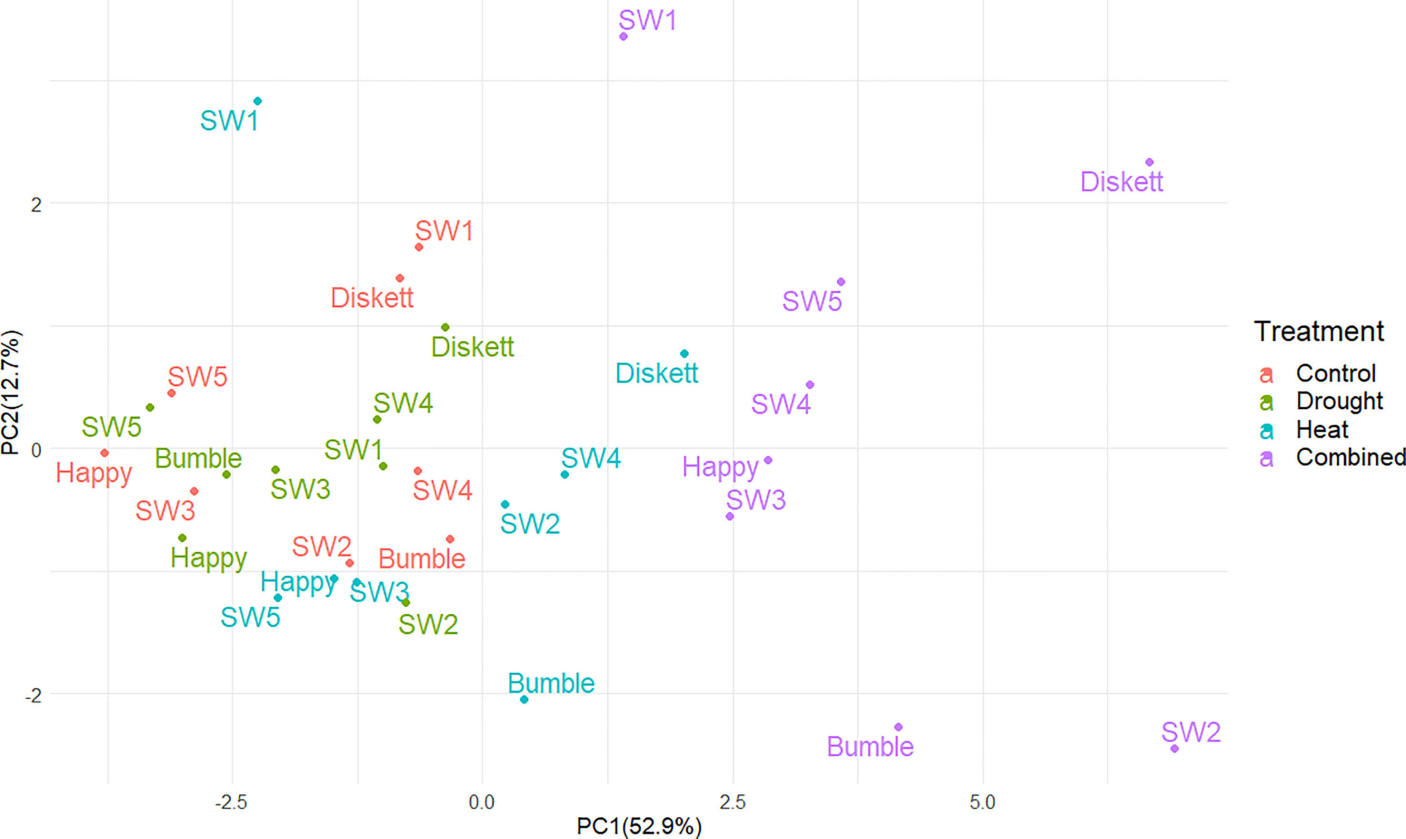
Principal component analysis (PCA) plot showing distribution of the eight wheat genotypes grown under control, drought, heat, and combined heat–drought stress conditions.

In PCA plots, the mean values of the phenotypic and gluten protein characteristics for plants in the four environments (treatments) explained 65.6% of the variation (PC1 52.9%, PC2 12.7%) (**Figure 4**). The strongest impact on the genotypes was observed in the combined heat–drought treatment, for genotypes Diskett and SW2 (**Figure 4**). Sensitivity to heat stress and heat–drought stress was observed for genotype SW1 (**Figure 4**).

**Figure 5:**
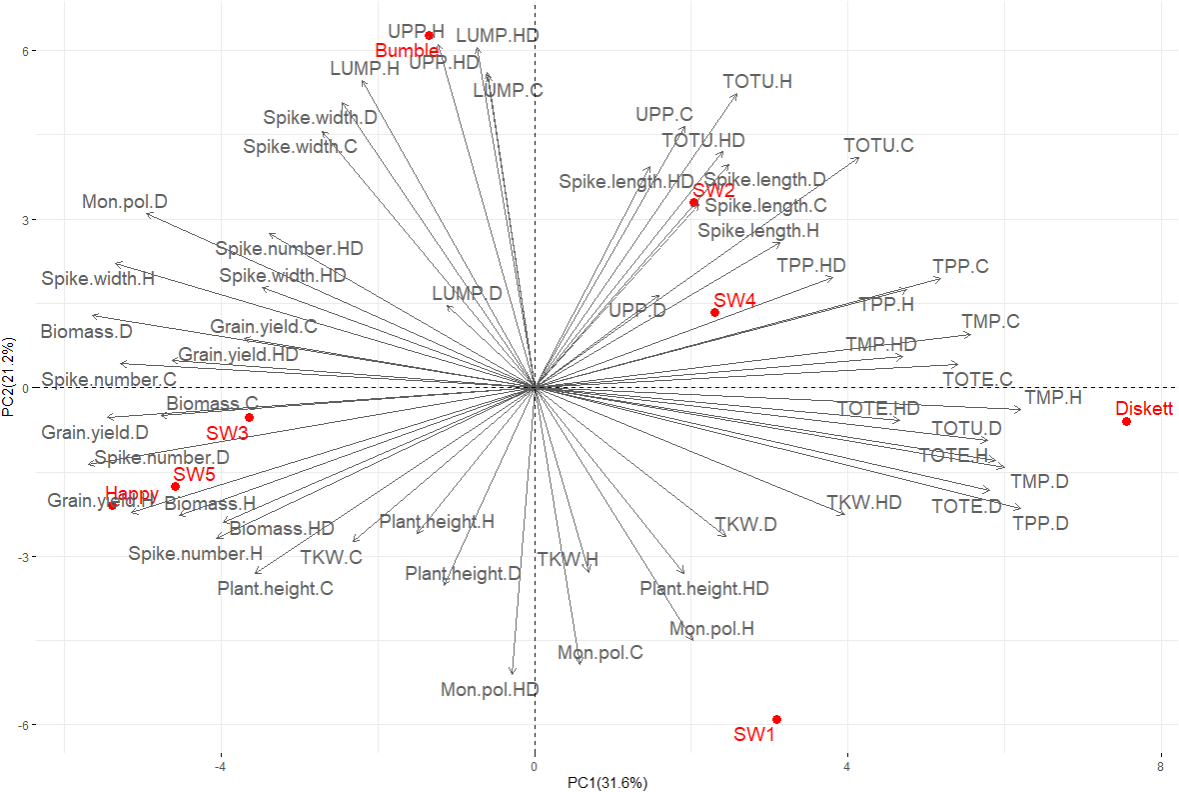
PCA plot showing the relationship between gluten protein parameters (TOTE, TOTU, TMP, TPP, %UPP, and %LUMP) evaluated by size-exclusion high-performance liquid chromatography and phenotypic traits [thousand kernel weight (TKW), plant height, spike length, biomass, spike width and grain yield] of the eight wheat genotypes grown under control (C), drought (D), heat (H), and combined heat–drought (HD) conditions.

Yield, phenotypic characteristics, and gluten protein parameters explained 52.8% of the variation in PCA (PC1 31.6%, PC2 21.2%) (**Figure 5**). The major contributors to these two PCs were %UPP (control and drought), TOTU (heat, heat–drought, and control), and spike length (all treatments), which impacted SW4 and SW2 most, further followed by Diskett. Happy, SW3, and SW5 showed a similar low response to the stresses, together with grain yield (all treatments) (**Figure 5**). Bumble displayed a similar response in spike width (all stress treatments and control), %LUMP (control, heat, and heat–drought), and %UPP (heat and heat–drought) (**Figure 5**).

**Figure 6:**
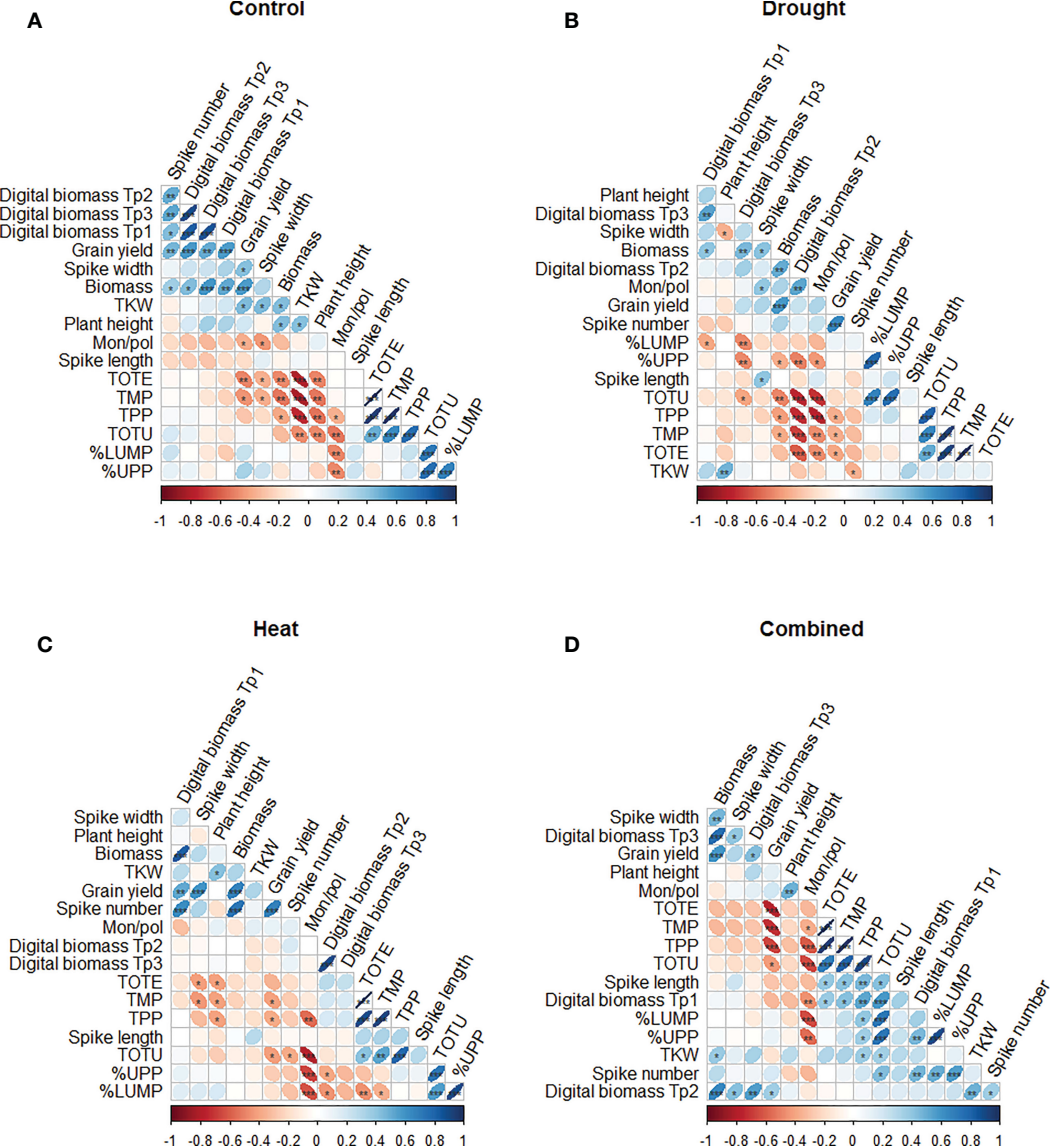
Spearman correlation plot of gluten protein parameters (TOTE, TOTU, TMP, TPP, %UPP and %LUMP) and phenotypic traits (grain yield, TKW, plant height, spike length, biomass, spike width, and the number of spikes) of the eight wheat genotypes grown under **(A)** control, **(B)** drought, **(C)** heat, and **(D)** combined heat–drought conditions. ***, **, and * indicate significance at p< 0.001, p< 0.01, and p< 0.05, respectively.

The Spearman’s rank correlation results indicated a significant impact of the treatments on certain gluten protein parameters, yield, and phenotypic traits (**Figure 6**). In the control (unstressed) environment, a significant positive correlation was found between grain yield and digital biomass at all three time points (Tp1–Tp3) (p< 0.001), and between grain yield and the number of spikes (p< 0.01) (**Figure 6**). Experimentally measured biomass showed a significant positive correlation with digital biomass at Tp1, Tp2, and Tp3 (p< 0.001, p< 0.05, and p< 0.001, respectively) (**Figure 6A**). The strongest significant negative correlations were found between TKW and the protein parameters [TOTE, TMP, TPP (p< 0.001); TOTU (p< 0.01)] (**Figure 6A**).

In all stress treatments, grain yield was significantly positively correlated with biomass (p< 0.001), while significant positive correlations were also found between grain yield and digital biomass under heat (p< 0.01) and under combined heat–drought stress (p< 0.05) (**Figures 6C, D**).

Under drought treatment, a negative significant correlation was found between digital biomass (at Tp2) and most of the protein parameters studied (TOTU, TPP TMP, and TOTE; p< 0.001) (**Figure 6B**). Additionally, TPP, TMP, and TOTE showed a significant negative correlation with grain yield (**Figure 6B**).

Under individual drought and heat stresses, a significant negative correlation between digital biomass (at Tp3 and Tp2) and %UPP (p< 0.01 and p< 0.05, respectively) was observed (**Figures 6B, C**). Under heat stress, only TKW was significantly positively correlated with plant height (p< 0.05).

In combined heat–drought stress conditions, a significant negative correlation was found between grain yield and protein parameters (TOTE, TMP, and TPP; p<0.001) (**Figure 6D**). TOTU showed significant positive correlations with digital biomass at Tp1 (p< 0.001) and with the phenotypic traits spike length, the number of spikes, and TKW (p< 0.05). In addition, the number of spikes was significantly positively correlated with %UPP (p< 0.001) and %LUMP (p< 0.01) (**Figure 6D**).

**Figure 7:**
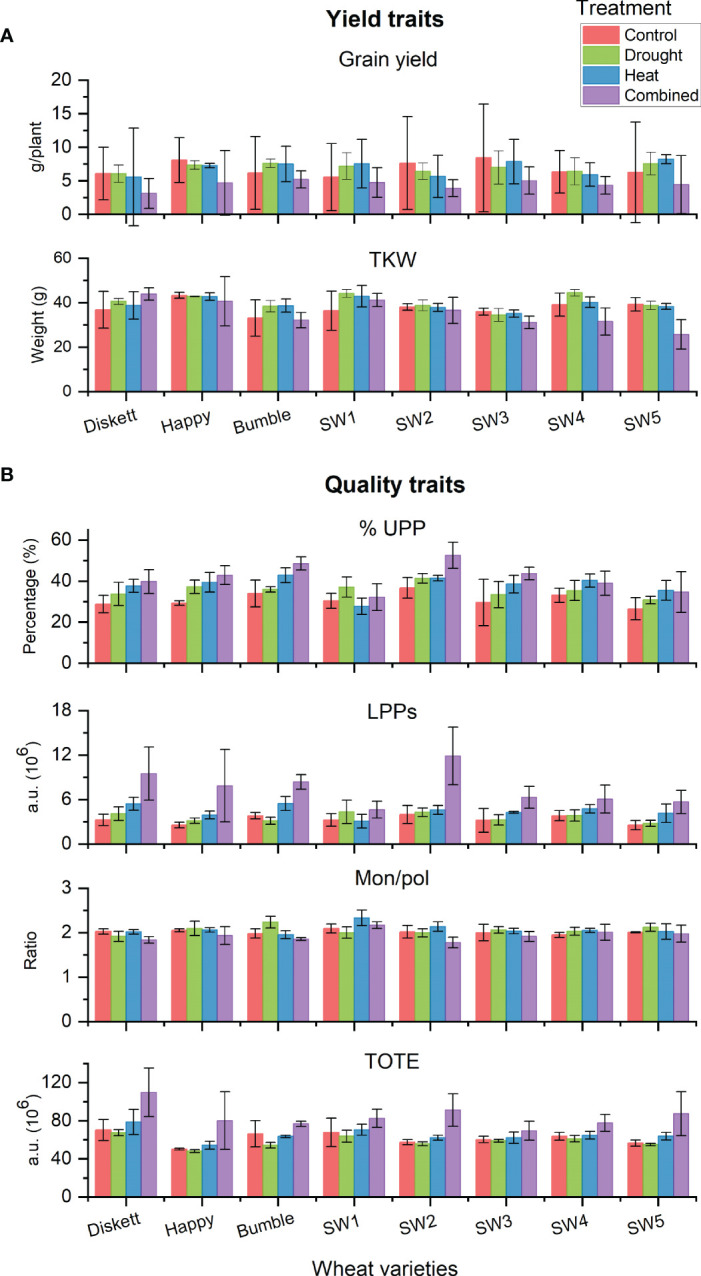
**(A)** Yield traits (grain yield and TKW) and **(B)** gluten protein traits (%UPP, LPPs, Mon/pol, and TOTE) of the eight individual wheat genotypes grown under control, drought, heat, and combined heat–drought conditions.

Comparisons of the impact of the stress treatments on selected yield traits (grain yield and TKW) and gluten parameters (%UPP, LPP, Mon/pol, and TOTE) for individual genotypes revealed some variation (non-significant) between the genotypes (**Figure 7**). Combined heat–drought stress decreased grain yield in most genotypes (**Figure 7A**). However, Bumble and SW1 in the combined heat–drought treatment showed similar grain yield as in the control. Regarding TKW, no impact of the stresses was observed for most genotypes, except a decrease due to combined heat–drought stress for SW3, SW4, and SW5 (**Figure 7A**).

In terms of gluten protein characteristics, a clear increase in both %UPP and LPPs (gluten strength) was noted for SW2, which had the highest %UPP (52.5%) of all genotypes studied (**Figure 7B**). Concerning the Mon/pol ratio (describing extensibility vs. strength distribution), no difference due to the stresses was found between the genotypes. Total extractable protein (TOTE), a strong indicator of protein concentration, was found to be increased most under combined heat–drought stress in genotype SW2 (**Figure 7B**).

### 3.4 Stability of yield and protein quality traits under stress

**Figure 8:**
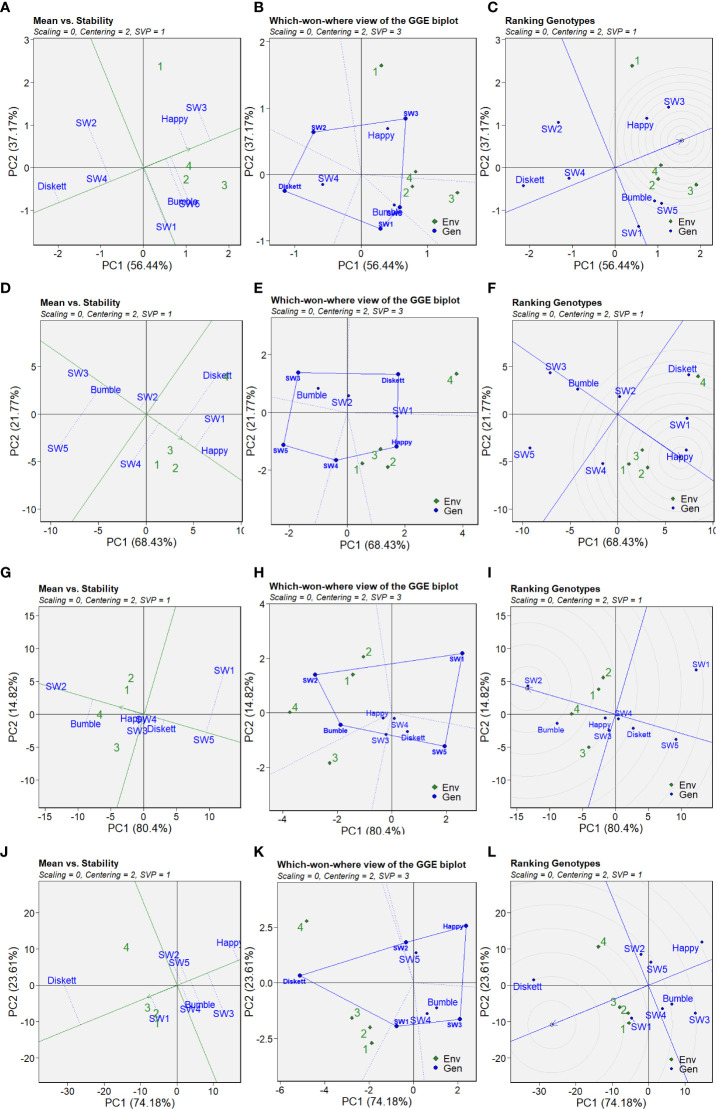
GGE biplots showing the stability of the wheat genotypes in the different environments studied: **(A–C)** grain yield, **(D–F)** TKW, **(G–I)** %UPP, and **(J–L)** TOTE (10^6^). Biplots from genotype-based singular value partitioning (SVP = 1); data were not scaled (scaling = 0) and were environment-centered (centering = 2). Environments 1, 2, 3, and 4 correspond to the control (no stress), drought, heat, and combined heat–drought stress treatments, respectively.

The GGE biplots of PC1 and PC2 scores indicating the stability and performance of the wheat genotypes in terms of grain yield, TKW, TOTE, and %UPP in the different treatments are shown in **Figure 8**. PC1 and PC2 together explained 93.61% of the variation in grain yield, 90.2% of the variation in TKW, 95.22% of the variation in %UPP (gluten strength), and 97.79% of the variation in TOTE (protein concentration) (**Figure 8**). To identify stable genotypes, the GGE biplots of mean performance and stability across the environments were compared, where 1, 2, 3, and 4 in **Figure 8** correspond to control, drought, heat, and combined heat–drought, respectively.

The grain yield (g/plant) plot for ‘mean vs. stability’ revealed the most promising genotypes (**Figure 8A**). SW3 showed the highest mean value for grain yield, followed by Happy, while Diskett and SW4 showed the lowest grain yield in the studied environments (**Figure 8A**). The five connected points in the ‘which-won-where’ GGE biplots showed that plants in the drought, heat, and combined heat–drought treatments clustered close to each other and indicated that the genotypes Bumble, SW5, Happy, and SW3 were the top performers in the studied environments (**Figure 8B**). Ranking the genotypes according to their location in the circles in the GGE biplots confirmed that the genotypes Happy and SW3 were the top performers, i.e., located closest to the “ideal line” (**Figure 8C**).

Stability in TKW appeared to be higher for the genotype Happy (**Figure 8D**). The genotypes Bumble and SW3 showed the highest stability, but the lowest mean values, for TKW (**Figure 8D**). The five connected points in the ‘which-won-where’ GGE biplots showed that the control, drought, and heat treatments (1, 2, and 3, respectively) clustered in the same section, with Happy and SW4 indicated as the top performers (**Figure 8E**). For the combined heat–drought environment, the top performer in terms of TKW was Diskett (**Figure 8E**). Based on its location in the inner circle and position near the “ideal line,” Happy was identified as the top-ranking genotype (**Figure 8F**).

The GGE biplots indicated that stability in %UPP among the genotypes in the studied environments was highest in terms of the mean value for SW2, followed by Bumble (**Figure 8G**). The ‘which-won-where’ GGE biplots indicated that SW2 was the top performer in the control, drought, and combined heat–drought treatments, while Bumble was the top performer in the heat and combined heat–drought treatments (**Figure 8H**). The highest ranking genotypes in all four environments were SW2 and Bumble (**Figure 8I**).

Stability evaluation of TOTE in the GGE biplots showed that the highest mean values across the studied environments were for Diskett and SW1 (**Figure 8J**). Based on the ‘which-won-where’ GGE biplots, Diskett was a top performer in all four environments, while SW1 was a top performer in the control, drought, and heat treatments (**Figure 8K**). SW2 and SW5 were the top performers in the combined heat–drought treatment (**Figure 8K**). To conclude, the top-ranked genotypes in all environments for TOTE were Diskett and SW1 (**Figure 8L**).

## 4 Discussion

Accurate tools for evaluating the yield, phenotypic traits, and quality traits of wheat genotypes under changing climate conditions are important when selecting new cultivars. Among the abiotic stresses to which plants are subjected, drought, heat, and combined heat–drought are major limiting factors affecting wheat plant development. In this study, combined heat–drought stress had the greatest impact on digital biomass (assessed by RGB imaging) in the eight wheat genotypes studied. The drought treatment also significantly reduced the digital biomass, whereas the heat stress treatment had a relatively mild impact (**Figures 1**, **2**). A similar pattern has been observed previously under combined heat–drought stress conditions for Nordic wheat grown in controlled conditions and Lithuanian winter wheat grown in the field (Statkevičiūtė et al., 2022). These observations suggest that the combined stress affects physiological plant traits such as stomatal closure, which leads to decreased CO_2_ assimilation and lower TKW (Abdelhakim et al., 2021; Statkevičiūtė et al., 2022). The magnitude of the reduction in biomass is known to depend on the duration and intensity of the stress and when the stress is imposed (Barnabás et al., 2008; Farooq et al., 2009; Qaseem et al., 2019).

In this study, wheat plants were exposed to the different 5-day stress treatments during anthesis, which is known to be one of the most critical growth stages, explaining the strong impact of the treatments involving drought. Applying heat stress alone had a mild impact on plant development characteristics and the biomass of individual genotypes; thus, it was not possible to evaluate plant response mechanisms to this stress. The chosen heat stress temperature (29°C) was based on findings in previous studies that a temperature of 27°C–30°C or higher prior to and during anthesis can substantially reduce grain size, numbers, and yield (Tashiro and Wardlaw, 1989; Wheeler et al., 1996; Porter and Semenov, 2005; Semenov and Shewry, 2011).

As expected, drought and combined heat–drought stress decreased biomass accumulation (**Figure 3**). The lack of impact of heat stress alone on biomass was most likely insufficiently high temperature and short treatment time resulting in little damage to photo-system II and thus to photosynthetic capacity (Sharkey, 2005), as seen for Bumble and SW2 at Tp2 (**Figure 3C**). For example, Diskett plants responded less to heat than to drought or combined heat–drought stress, despite the PCA results indicating some sensitivity of this genotype to heat stress (**Figure 4**). This can be explained by a different response mechanism of Diskett to heat and heat–drought stresses, as referred to our previous study (Lama et al., 2022). For a detailed examination of the heat response of Diskett, field studies under heat stress conditions are needed.

The digital tools used in this study to measure digital biomass showed good ability to evaluate wheat plants under severe drought and combined heat–drought stress conditions. The correlations observed between digital and measured biomass, e.g., under drought conditions (at Tp3) and combined heat–drought conditions (at Tp2), revealed the strong potential of RGB imaging to identify even small differences induced by stresses and to detect symptoms of the genotypes under the different environments. Under heat stress, the genotypes SW1 and SW3 were least affected (**Figure 3C**), while under combined heat–drought stress, Happy and SW3 were the least affected genotypes (**Figure 3D**), suggesting somewhat different stress coping mechanisms.

In the control (no stress) growing environment, most gluten parameters (TOTE, etc.) showed a negative correlation with grain yield, TKW, biomass, plant height, and spike width (**Figure 6A**), as also observed previously (Bogard et al., 2011; Wang et al., 2018). TKW and grain yield are closely associated (Li et al., 2021) and are linked with starch accumulation. High accumulation of starch under the control conditions can dilute the gluten protein concentrations in wheat grain (Koga et al., 2015), which may be one explanation for the negative correlations between gluten proteins and yield-related parameters in this study.

This study examined the impact of stress factors on the most important gluten quality parameters, e.g., gluten strength, based on the concentrations of polymeric proteins. Under individual drought and heat stress, a negative correlation between %UPP and digital biomass (Tp2–Tp3) was observed. There was also a clear impact of stress on the %UPP and %LUPP fractions (**Table 3**), suggesting that these environmental stresses trigger mechanisms related to gluten polymer accumulation/regulation. In the presence of severe stress (combined heat–drought treatment), the wheat plants seemed still able to produce spikes, as the number of spikes correlated positively with %UPP.

Spike length is a strong indicator of yield (Lan et al., 2022) and is directly related to starch accumulation, suggesting that wheat yield and protein polymerization are in some way related. Under stress conditions, gluten polymerization is triggered via the formation of interchain disulfide bonds (SS) between HMW and LMW glutenins and certain gliadins (alpha, beta, and gamma) (Branlard et al., 2020). These gluten proteins form %UPP most likely at the expense of starch. Previous studies have found that, in particular, large polymeric protein fractions (e.g., uLPP and uSPP) increase at 12–18 days after anthesis (Johansson et al., 2005). Under combined heat–drought stress conditions in the present study, yield was significantly negatively correlated with protein concentration, confirming findings in previous studies in the field and greenhouse (Triboi et al., 2006; Malik et al., 2012).

A positive effect of abiotic stress on gluten protein polymerization has also been observed in previous studies performed in the field (Johansson, 2002; Johansson et al., 2002; Lama et al., 2022) and greenhouse (Malik et al., 2011; Leiva et al., 2021). The positive effect of heat stress on protein polymerization is known to occur at high temperatures (up to 30°C) during the grain development stage (Johansson et al., 2002; Malik et al., 2011). The nature of the effect of drought stress on polymerization depends on the timing of the drought (Leiva et al., 2021; Lan et al., 2022). For example, a greenhouse study found a positive effect of drought at heading, which increased %UPP compared with drought at the stem elongation stage (Leiva et al., 2021). Late drought (during ear emergence) is reported to have a stronger positive effect on %UPP than early drought (during tillering) (Lan et al., 2022). Among the three stress treatments tested in this study, combined heat–drought stress had the greatest effect on gluten protein polymerization.

The results obtained with the imaging tools employed to measure digital biomass at different stress time points (Tp1, Tp2, and Tp3) in this study were significantly and positively correlated with grain yield and with actual measured biomass, suggesting that RGB imaging could be a useful method for evaluating the impacts of plant stresses on phenotypic characteristics, such as grain yield.

No significant effect was found for the interaction between the genotype and the environment (G × E) on gluten protein parameters (except for Mon/pol), contradicting findings in previous studies in the greenhouse (Malik et al., 2013) and in the field (Hernandez-Espinosa et al., 2018; Lama et al., 2022). This lack of effect may have been due to the insufficiently challenging environmental background in this study.

Among the eight genotypes compared, Happy was the most promising in terms of the stability of grain yield, TKW, %UPP, and TOTE in stressful growing environments. Happy also showed greater digital biomass in the combined heat–drought stress treatment. The breeding line SW2 showed not only lower digital biomass and yield under stress but also the highest stability and mean gluten strength (%UPP) in the control and in all three stress treatments, supporting previous findings (Lama et al., 2022). Genotype SW3 showed the highest stability in TKW and higher grain yield than the other genotypes. Diskett, SW5, and SW1 appeared to be the most sensitive genotypes in terms of most parameters studied (yield, TKW, %UPP, etc.).

## 5 Conclusions

Assessing and controlling important phenotypic and grain quality–related traits in wheat is important for success in breeding programs seeking to produce desirable wheat material for use under future climate change. This study revealed significant impacts of combined heat–drought stress on plant phenotypic characteristics and gluten protein quality traits in eight spring wheat genotypes, while drought stress alone also had negative impacts. However, heat stress (29°C) had only mild effects on yield and phenotypic characteristics, although, in field conditions, the impact of heat stress could be much more severe.

There were significant positive correlations between grain yield and digital biomass, and between digital biomass and actual measured biomass, in all stress treatments tested, indicating that RGB imaging can be a valuable tool in assessing stress in wheat plants.

Individual drought and heat stresses significantly affected gluten strength (%UPP and %LUPP). There was a negative correlation between digital biomass and most gluten protein parameters analyzed, although the Mon/pol ratio was not affected by the experimental stresses studied.

A surprising finding was that the number of spikes was significantly positively correlated with both %UPP and %LUMP under combined heat–drought stress, suggesting a correlation with not only polymeric glutenins (HMW and LMW) but also large monomeric proteins (e.g., gliadin types). The number of spikes is an indicator of yield and, together with gluten protein quality traits, could potentially be explored in screening for high yield and gluten protein quality in wheat under climate stress.

The most promising genotypes in terms of performance and stability in the stress environments tested were SW3 and Happy for high yield, SW1 and Happy for high TKW, SW2 and Bumble for high %UPP, and Diskett and SW1 for high protein concentration (TOTE). In order to meet plant breeding targets for extreme climate resistance, these top-performing genotypes need to be further tested in field studies where their phenotypic and gluten protein characteristics are evaluated using the combination of tools tested in this study.

## Data availability statement

The original contributions presented in the study are included in the article/**Supplementary Material** further inquiries can be directed to the corresponding author/s.

## Author contributions

RK and AC conceived the study. RK, AC, SL, and FL planned the greenhouse experiments. PV developed the breeding population set. SL and FL performed the greenhouse experiments, analyzed the data, and wrote the manuscript with inputs from RK and AC. RK acquired the funding. All authors contributed to data interpretation and approved the final version of the manuscript.

## References

[B1] Abdelhakim L. O. A. Rosenqvist E. Wollenweber B. Spyroglou I. Ottosen C.-O. Panzarová K. (2021). Investigating combined drought- and heat stress effects in wheat under controlled conditions by dynamic image-based phenotyping. Agronomy 11 (2), 364. doi: 10.3390/agronomy11020364

[B2] Anjum S. Xie X.-y. Wang L.-c. Saleem M. Man C. Lei W. (2011). Morphological, physiological and biochemical responses of plants to drought stress. Afr. J. Agric. Res. 6, 2026–2032. doi: 10.5897/AJAR10.027

[B3] Armoniené R. Odilbekov F. Vivekanand V. Chawade A. (2018). Affordable imaging lab for noninvasive analysis of biomass and early vigour in cereal crops. BioMed. Res. Int. 2018, 1–9. doi: 10.1155/2018/5713158 PMC5933035 29850536

[B4] Asseng S. Martre P. Maiorano A. Rötter R. P. O’leary G. J. Fitzgerald G. J.. (2019). Climate change impact and adaptation for wheat protein. Glob. Change Biol. 25 (1), 155–173. doi: 10.1111/gcb.14481 30549200

[B5] Barnabás B. Jäger K. Fehér A. (2008). The effect of drought and heat stress on reproductive processes in cereals. Plant Cell Environ. 31, 11–38. doi: 10.1111/j.1365-3040.2007.01727.x 17971069

[B6] Blum A. Sullivan C. Y. Nguyen H. T. (1997). The effect of plant size on wheat response to agents of drought stress. II. water deficit, heat and ABA. Funct. Plant Biol. 24 (1), 43–48. doi: 10.1071/PP96023

[B7] Bogard M. Jourdan M. Allard V. Martre P. Perretant M. R. Ravel C.. (2011). Anthesis date mainly explained correlations between post-anthesis leaf senescence, grain yield, and grain protein concentration in a winter wheat population segregating for flowering time QTLs. J. Exp. Bot. 62 (10), 3621–3636. doi: 10.1093/jxb/err061 21414962

[B8] Branlard G. Faye A. Rhazi L. Tahir A. Lesage V. Aussenac T. (2020). Genetic and environmental factors associated to glutenin polymer characteristics of wheat. Foods 9 (5), 683. doi: 10.3390/foods9050683 32466243 PMC7278847

[B9] Cao Z. Yao X. Liu H. Liu B. Cheng T. Tian Y.. (2019). Comparison of the abilities of vegetation indices and photosynthetic parameters to detect heat stress in wheat. Agric. For. Meteorol. 265, 121–136. doi: 10.1016/j.agrformet.2018.11.009

[B10] Chawade A. van Ham J. Blomquist H. Bagge O. Alexandersson E. Ortiz R. (2019). High-throughput field-phenotyping tools for plant breeding and precision agriculture. Agronomy 9 (5), 258. doi: 10.3390/agronomy9050258

[B11] Daniel C. Triboi E. (2000). Effects of temperature and nitrogen nutrition on the grain composition of winter wheat: effects on gliadin content and composition. J. Cereal Sci. 32 (1), 45–56. doi: 10.1006/jcrs.2000.0313

[B12] Dhondt S. Wuyts N. Inzé D. (2013). Cell to whole-plant phenotyping: the best is yet to come. Trends Plant Sci. 18 (8), 428–439. doi: 10.1016/j.tplants.2013.04.008 23706697

[B13] Easlon H. M. Bloom A. J. (2014). Easy leaf area: automated digital image analysis for rapid and accurate measurement of leaf area. Appl. Plant Sci. 2 (7), 1–4. doi: 10.3732/apps.1400033 PMC4103476 25202639

[B14] EEA (European Environment Agency). (2019). Climate change adaptation in the agriculture sector in Europe. EEA Report No 4/2019. Copenhagen, Denmark. Available at: https://www.eea.europa.eu/highlights/climate-change-threatens-future-of.

[B15] Farooq M. Wahid A. Kobayashi N. Fujita D. Basra S. M. A. (2009). Plant drought stress: effects, mechanisms and management. Agron. Sustain. Dev. 29 (1), 185–212. doi: 10.1051/agro:2008021

[B16] Guzmán C. Autrique J. E. Mondal S. Singh R. P. Govindan V. Morales-Dorantes A.. (2016). Response to drought and heat stress on wheat quality, with special emphasis on bread-making quality, in durum wheat. Field Crops Res. 186, 157–165. doi: 10.1016/j.fcr.2015.12.002

[B17] Hernandez-Espinosa N. Mondal S. Autrique E. Gonzalez-Santoyo H. Crossa J. Huerta-Espino J.. (2018). Milling, processing and end-use quality traits of CIMMYT spring bread wheat germplasm under drought and heat stress. Field Crops Res. 215, 104–112. doi: 10.1016/j.fcr.2017.10.003

[B18] Humplík J. F. Lazár D. Husičková A. Spíchal L. (2015). Automated phenotyping of plant shoots using imaging methods for analysis of plant stress responses – a review. Plant Methods 11 (1), 29. doi: 10.1186/s13007-015-0072-8 25904970 PMC4406171

[B19] Hurkman W. J. Wood D. F. (2011). High temperature during grain fill alters the morphology of protein and starch deposits in the starchy endosperm cells of developing wheat (Triticum aestivum l.) grain. J. Agric. Food Chem. 59 (9), 4938–4946. doi: 10.1021/jf102962t 21417450

[B20] Islas-Rubio A. R. Singh H. Chittrakorn S. MacRitchie F. (2006). Stability of wheat proteins in solution. J. Cereal Sci. 43 (2), 169–174. doi: 10.1016/j.jcs.2005.08.009

[B21] Istvan D. (2014). DigiCamControl Software, Version 2.1.2. [(accessed on 15 December 2022)]. Available online: http://digicamcontrol.com/.

[B22] Johansson E. (2002). Effect of two wheat genotypes and Swedish environment on falling number, amylase activities, and protein concentration and composition. Euphytica 126 (1), 143–149. doi: 10.1023/A:1019646916905

[B23] Johansson E. Branlard G. Cuniberti M. Flagella Z. Hüsken A. Nurit E.. (2020). “Genotypic and environmental effects on wheat technological and nutritional quality,” in Wheat quality for improving processing and human health (Springer), 171–204. doi: 10.1007/978-3-030-34163-3_8

[B24] Johansson E. Kuktaite R. Andersson A. Prieto-Linde M. L. (2005). Protein polymer build-up during wheat grain development: influences of temperature and nitrogen timing. J. Sci. Food Agric. 85 (3), 473–479. doi: 10.1002/jsfa.2006

[B25] Johansson E. Nilsson H. Mazhar H. Skerritt J. MacRitchie F. Svensson G. (2002). Seasonal effects on storage proteins and gluten strength in four Swedish wheat cultivars. J. Sci. Food Agric. 82, 1305–1311. doi: 10.1002/jsfa.1185

[B26] Koga S. Böcker U. Moldestad A. Tosi P. Shewry P. R. Mosleth E. F.. (2015). Influence of temperature on the composition and polymerization of gluten proteins during grain filling in spring wheat (Triticum aestivum l.). J. Cereal Sci. 65, 1–8. doi: 10.1016/j.jcs.2015.05.012

[B27] Kuktaite R. Larsson H. Johansson E. (2004). Variation in protein composition of wheat flour and its relationship to dough mixing behaviour. J. Cereal Sci. 40 (1), 31–39. doi: 10.1016/j.jcs.2004.04.007

[B28] Kumar D. Kushwaha S. Delvento C. Liatukas Ž. Vivekanand V. Svensson J. T.. (2020). Affordable phenotyping of winter wheat under field and controlled conditions for drought tolerance. Agronomy 10 (6), 882. doi: 10.3390/agronomy10060882

[B29] Lama S. Vallenback P. Hall S. A. Kuzmenkova M. Kuktaite R. (2022). Prolonged heat and drought versus cool climate on the Swedish spring wheat breeding lines: impact on the gluten protein quality and grain microstructure. Food Energy Secur. 11 (2), 1–17. doi: 10.1002/fes3.376

[B30] Lan Y. Chawade A. Kuktaite R. Johansson E. (2022). Climate change impact on wheat performance - effects on vigour, plant traits and yield from early and late drought stress in diverse lines. Int. J. Mol. Sci. 23 (6), 1–17. doi: 10.3390/ijms23063333 PMC8950129 35328754

[B31] Le Gouis J. Oury F.-X. Charmet G. (2020). How changes in climate and agricultural practices influenced wheat production in Western Europe. J. Cereal Sci. 93, 102960. doi: 10.1016/j.jcs.2020.102960

[B32] Leiva F. Vallenback P. Ekblad T. Johansson E. Chawade A. (2021). Phenocave: an automated, standalone, and affordable phenotyping system for controlled growth conditions. Plants 10 (9), 1817. doi: 10.3390/plants10091817 34579350 PMC8469120

[B33] Li S. Wang L. Meng Y. Hao Y. Xu H. Hao M.. (2021). Dissection of genetic basis underpinning kernel weight-related traits in common wheat. Plants 10 (4), 1–14. doi: 10.3390/plants10040713 PMC8103506 33916985

[B34] Malik A. H. Andersson A. Kuktaite R. Mujahid M. Y. Khan B. Johansson E. (2012). Genotypic variation in dry weight and nitrogen concentration of wheat plant parts; relations to grain yield and grain protein concentration. J. Agric. Sci. 4 (11), 11–15. doi: 10.5539/jas.v4n11p11

[B35] Malik A. H. Kuktaite R. Johansson E. (2013). Combined effect of genetic and environmental factors on the accumulation of proteins in the wheat grain and their relationship to bread-making quality. J. Cereal Sci. 57 (2), 170–174. doi: 10.1016/j.jcs.2012.09.017

[B36] Malik A. H. Prieto-Linde M. L. Kuktaite R. Andersson A. Johansson E. (2011). Individual and interactive effects of cultivar maturation time, nitrogen regime and temperature level on accumulation of wheat grain proteins. J. Sci. Food Agric. 91 (12), 2192–2200. doi: 10.1002/jsfa.4439 21547918

[B37] Mamrutha H. M. Khobra R. Sendhil R. Munjal R. Sai Prasad S. V. Biradar S.. (2020). Developing stress intensity index and prioritizing hotspot locations for screening wheat genotypes under climate change scenario. Ecol. Indic. 118, 106714. doi: 10.1016/j.ecolind.2020.106714

[B38] Peña R. J. (2007). Current and future trends of wheat quality needs (Dordrecht: Springer Netherlands).

[B39] Porter J. R. Semenov M. A. (2005). Crop responses to climatic variation. Philos. Trans. R. Soc B: Biol. Sci. 360 (1463), 2021–2035. doi: 10.1098/rstb.2005.1752 PMC1569569 16433091

[B40] Prasad P. V. V. Pisipati S. R. Momčilović I. Ristic Z. (2011). Independent and combined effects of high temperature and drought stress during grain filling on plant yield and chloroplast EF-tu expression in spring wheat. J. Agron. Crop Sci. 197 (6), 430–441. doi: 10.1111/j.1439-037X.2011.00477.x

[B41] Qaseem M. F. Qureshi R. Shaheen H. (2019). Effects of pre-anthesis drought, heat and their combination on the growth, yield and physiology of diverse wheat (Triticum aestivum l.) genotypes varying in sensitivity to heat and drought stress. Sci. Rep. 9 (1), 6955. doi: 10.1038/s41598-019-43477-z 31061444 PMC6502848

[B42] Rampino P. Pataleo S. Gerardi C. Mita G. Perrotta C. (2006). Drought stress response in wheat: physiological and molecular analysis of resistant and sensitive genotypes. Plant Cell Environ. 29 (12), 2143–2152. doi: 10.1111/j.1365-3040.2006.01588.x 17081248

[B43] Reynolds D. Baret F. Welcker C. Bostrom A. Ball J. Cellini F.. (2019). What is cost-efficient phenotyping? optimizing costs for different scenarios. Plant Sci. 282, 14–22. doi: 10.1016/j.plantsci.2018.06.015 31003607

[B44] Rizhsky L. Liang H. Mittler R. (2002). The combined effect of drought stress and heat shock on gene expression in tobacco. Plant Physiol. 130 (3), 1143–1151. doi: 10.1104/pp.006858 12427981 PMC166635

[B45] Sara D. Mandava A. K. Kumar A. Duela S. Jude A. (2021). Hyperspectral and multispectral image fusion techniques for high resolution applications: a review. Earth Sci. Inform. 14 (4), 1685–1705. doi: 10.1007/s12145-021-00621-6

[B46] Sattar A. Sher A. Ijaz M. Ullah M. S. Ahmad N. Umar U. U.-D. (2020). Individual and combined effect of terminal drought and heat stress on allometric growth, grain yield and quality of bread wheat. Pak. J. Bot. 52 (2), 405–412. doi: 10.30848/pjb2020-2(5

[B47] Semenov M. A. Shewry P. R. (2011). Modelling predicts that heat stress, not drought, will increase vulnerability of wheat in Europe. Sci. Rep. 1 (1), 66. doi: 10.1038/srep00066 22355585 PMC3216553

[B48] Sharkey T. D. (2005). Effects of moderate heat stress on photosynthesis: importance of thylakoid reactions, rubisco deactivation, reactive oxygen species, and thermotolerance provided by isoprene. Plant Cell Environ. 28 (3), 269–277. doi: 10.1111/j.1365-3040.2005.01324.x

[B49] Shew A. M. Tack J. B. Nalley L. L. Chaminuka P. (2020). Yield reduction under climate warming varies among wheat cultivars in south Africa. Nat. Commun. 11 (1), 4408. doi: 10.1038/s41467-020-18317-8 32879311 PMC7468144

[B50] Shiferaw B. Smale M. Braun H.-J. Duveiller E. Reynolds M. Muricho G. (2013). Crops that feed the world 10. past successes and future challenges to the role played by wheat in global food security. Food Secur. 5 (3), 291–317. doi: 10.1007/s12571-013-0263-y

[B51] Singh R. P. Prasad P. V. V. Sunita K. Giri S. N. Reddy K. R. (2007). Influence of high temperature and breeding for heat tolerance in cotton: a review. Adv. Agron. 93, 313–385. doi: 10.1016/S0065-2113(06)93006-5

[B52] Statkevičiūtė G. Liatukas Ž. Cesevičienė J. Jaškūnė K. Armonienė R. Kuktaite R.. (2022). Impact of combined drought and heat stress and nitrogen on winter wheat productivity and end-use quality. Agronomy 12 (6), 1452. doi: 10.3390/agronomy12061452

[B53] Tao H. Xu S. Tian Y. Li Z. Ge Y. Zhang J.. (2022). Proximal and remote sensing in plant phenomics: 20 years of progress, challenges, and perspectives. Plant Commun. 3 (6), 100344. doi: 10.1016/j.xplc.2022.100344 35655429 PMC9700174

[B54] Tashiro T. Wardlaw I. F. (1989). A comparison of the effect of high-temperature on grain development in wheat and rice. Ann. Bot. 64, 59–65. doi: 10.1093/oxfordjournals.aob.a087808

[B55] Team R. C. (2013). R: a language and environment for statistical computing. Vienna, Austria: R Foundation for Statistical Computing.

[B56] Triboi E. Martre P. Girousse C. Ravel C. Triboi-Blondel A. (2006). Unravelling environmental and genetic relationships between grain yield and nitrogen concentration for wheat. Eur. J. Agron. 25 (2), 108–118. doi: 10.1016/j.eja.2006.04.004

[B57] Wahid A. Gelani S. Ashraf M. Foolad M. R. (2007). Heat tolerance in plants: an overview. Environ. Exp. Bot. 61 (3), 199–223. doi: 10.1016/j.envexpbot.2007.05.011

[B58] Wang X. Hou L. Lu Y. Wu B. Gong X. Liu M.. (2018). Metabolic adaptation of wheat grain contributes to a stable filling rate under heat stress. J. Exp. Bot. 69 (22), 5531–5545. doi: 10.1093/jxb/ery303 30476278 PMC6255704

[B59] Wheeler T. R. Hong T. D. Ellis R. H. Batts G. R. Morison J. I. L. Padley P. (1996). The duration and rate of grain growth and harvest index, of wheat (Triticum aestivum l) in response to temperature and CO_2_ . J. Exp.Bot. 47, 623–630. doi: 10.1093/jxb/47.5.623

[B60] Wu W. Zhou L. Chen J. Qiu Z. He Y. (2018). Gain TKW: a measurement system of thousand kernel weight based on the android platform. Agronomy 8 (9), 178. doi: 10.3390/agronomy8090178

[B61] Yadav S. Modi P. Dave A. Vijapura A. Patel D. Patel M. (2020). “Effect of abiotic stress in crops,” in Sustainable crop production. (London, UK: IntechOpen).

[B62] Zadoks J. C. Chang T. T. Konzak C. F. (1974). A decimal code for the growth stages of cereals. Weed Res. 14, 415–421. doi: 10.1111/j.1365-3180.1974.tb01084.x

[B63] Zhang T. He Y. DePauw R. Jin Z. Garvin D. Yue X.. (2022). Climate change may outpace current wheat breeding yield improvements in north America. Nat. Commun. 13 (1), 5591. doi: 10.1038/s41467-022-33265-1 36180462 PMC9525655

[B64] Zhao K. Tao Y. Liu M. Yang D. Zhu M. Ding J.. (2022). Does temporary heat stress or low temperature stress similarly affect yield, starch, and protein of winter wheat grain during grain filling? J. Cereal Sci. 103, 1–10. doi: 10.1016/j.jcs.2021.103408

[B65] Zhou R. Yu X. Ottosen C. O. Rosenqvist E. Zhao L. Wang Y.. (2017). Drought stress had a predominant effect over heat stress on three tomato cultivars subjected to combined stress. BMC Plant Biol. 17 (1), 24. doi: 10.1186/s12870-017-0974-x 28122507 PMC5264292

[B66] Zubler A. V. Yoon J.-Y. (2020). Proximal methods for plant stress detection using optical sensors and machine learning. Biosensors 10 (12), 193. doi: 10.3390/bios10120193 33260412 PMC7760370

