# Supplementary material for: Impacts of heat, drought, and combined heat–drought stress on yield, phenotypic traits, and gluten protein traits: capturing stability of spring wheat in excessive environments
Source: Front Plant Sci. 2023 May 19;14:1179701. doi: 10.3389/fpls.2023.1179701 (PMC10235758; doi:10.3389/fpls.2023.1179701)
Supplement: Supplementary file 1 [file Table_1.docx]

Supplementary Material

Article Title

Sbatie Lama^†^, Fernanda Leiva^1†^, Pernilla Vallenback^2^, Aakash Chawade^1^ and Ramune Kuktaite^1^

^1^Department of Plant Breeding, Swedish University of Agricultural Sciences, SE-23422 Lomma, Sweden

^2^Lantmännen Lantbruk, SE-26881 Svalöv, Sweden

†These authors contributed equally to this work.

*** Correspondence:** Corresponding Author: sbatie.lama@slu.se

# Supplementary Tables

**Table S1**. Climate data used in this study for heat and combined (heat and drought) treatments

*Average climate data of every 7 days during the wheat growing period (April 22nd -Aug 11th) over the past 5 years (2016–2020) in Malmö, Sweden used in this study.

**Temperature (℃)**

|  | | Hours | | | | | | | | | | | | | | | | | | | | | | | |
| --- | --- | --- | --- | --- | --- | --- | --- | --- | --- | --- | --- | --- | --- | --- | --- | --- | --- | --- | --- | --- | --- | --- | --- | --- | --- |
| Dates* | Number  of the  days | 0000 | 0100 | 0200 | 0300 | 0400 | 0500 | 0600 | 0700 | 0800 | 0900 | 1000 | 1100 | 1200 | 1300 | 1400 | 1500 | 1600 | 1700 | 1800 | 1900 | 2000 | 2100 | 2200 | 2300 |
| April 22 - 28 | 7 | 6 | 6 | 6 | 6 | 6 | 7 | 8 | 9 | 10 | 11 | 12 | 12 | 12 | 13 | 12 | 12 | 12 | 11 | 9 | 9 | 8 | 8 | 7 | 7 |
| April 29 - May 5 | 7 | 6 | 6 | 6 | 6 | 6 | 7 | 9 | 10 | 10 | 11 | 12 | 12 | 13 | 13 | 13 | 13 | 12 | 11 | 10 | 9 | 8 | 7 | 7 | 6 |
| May 6 - 12 | 7 | 8 | 7 | 7 | 7 | 8 | 9 | 11 | 12 | 13 | 14 | 15 | 16 | 16 | 16 | 16 | 16 | 15 | 14 | 13 | 11 | 10 | 9 | 9 | 8 |
| May 13 - 19 | 7 | 9 | 9 | 8 | 8 | 9 | 10 | 12 | 13 | 14 | 15 | 16 | 16 | 17 | 17 | 17 | 17 | 16 | 15 | 15 | 13 | 12 | 11 | 10 | 10 |
| May 20 - 26 | 7 | 11 | 11 | 11 | 10 | 11 | 13 | 15 | 16 | 17 | 17 | 18 | 19 | 19 | 19 | 19 | 19 | 18 | 18 | 17 | 15 | 14 | 13 | 12 | 12 |
| May 27 - June 2 | 7 | 13 | 12 | 12 | 12 | 13 | 15 | 16 | 17 | 18 | 19 | 20 | 21 | 21 | 21 | 21 | 21 | 20 | 20 | 18 | 17 | 15 | 14 | 14 | 13 |
| June 3 - June 9 | 7 | 13 | 12 | 12 | 12 | 13 | 15 | 16 | 17 | 19 | 19 | 20 | 20 | 21 | 21 | 21 | 20 | 20 | 19 | 18 | 17 | 15 | 14 | 14 | 13 |
| June 10 - 16 | 7 | 14 | 13 | 13 | 13 | 14 | 15 | 16 | 17 | 18 | 19 | 19 | 19 | 20 | 20 | 20 | 20 | 19 | 18 | 18 | 17 | 16 | 15 | 15 | 14 |
| June 17 - 23 | 7 | 15 | 14 | 14 | 14 | 15 | 16 | 17 | 18 | 19 | 19 | 20 | 20 | 21 | 21 | 21 | 21 | 20 | 20 | 19 | 18 | 17 | 16 | 16 | 15 |
| **Heat** | **5** | **29** | **29** | **29** | **29** | **29** | **29** | **29** | **29** | **29** | **29** | **29** | **29** | **29** | **29** | **29** | **29** | **29** | **29** | **29** | **29** | **29** | **29** | **29** | **29** |
| June 24 - 30 | 2 | 15 | 15 | 14 | 14 | 16 | 17 | 18 | 19 | 20 | 21 | 21 | 22 | 22 | 22 | 23 | 22 | 22 | 21 | 20 | 19 | 18 | 16 | 16 | 15 |
| July 1 - 7 | 7 | 14 | 14 | 14 | 14 | 14 | 15 | 16 | 16 | 17 | 17 | 18 | 19 | 19 | 19 | 19 | 19 | 18 | 18 | 18 | 17 | 16 | 15 | 15 | 15 |
| July 8 - 14 | 7 | 15 | 14 | 14 | 13 | 14 | 15 | 16 | 17 | 18 | 19 | 19 | 20 | 20 | 20 | 20 | 20 | 20 | 19 | 19 | 17 | 16 | 16 | 15 | 15 |
| July 15 - 21 | 7 | 15 | 15 | 14 | 14 | 15 | 16 | 18 | 19 | 19 | 20 | 20 | 21 | 21 | 21 | 21 | 21 | 21 | 20 | 20 | 18 | 17 | 16 | 16 | 15 |
| July 22 - 28 | 7 | 16 | 16 | 15 | 15 | 16 | 18 | 19 | 20 | 21 | 22 | 23 | 23 | 23 | 24 | 24 | 23 | 23 | 22 | 21 | 20 | 19 | 18 | 17 | 17 |
| July 29 - Aug 4 | 7 | 16 | 16 | 15 | 15 | 16 | 17 | 18 | 19 | 20 | 21 | 21 | 22 | 22 | 22 | 22 | 22 | 22 | 21 | 20 | 19 | 18 | 17 | 17 | 16 |
| Aug 5 - 11 | 7 | 17 | 16 | 16 | 16 | 16 | 17 | 18 | 20 | 21 | 21 | 22 | 23 | 23 | 23 | 23 | 22 | 22 | 21 | 20 | 19 | 18 | 18 | 17 | 17 |

**Relative humidity (%)**

|  | | Hours | | | | | | | | | | | | | | | | | | | | | | | |
| --- | --- | --- | --- | --- | --- | --- | --- | --- | --- | --- | --- | --- | --- | --- | --- | --- | --- | --- | --- | --- | --- | --- | --- | --- | --- |
| Dates* | Number of the days | 0000 | 0100 | 0200 | 0300 | 0400 | 0500 | 0600 | 0700 | 0800 | 0900 | 1000 | 1100 | 1200 | 1300 | 1400 | 1500 | 1600 | 1700 | 1800 | 1900 | 2000 | 2100 | 2200 | 2300 |
| April 22 - 28 | 7 | 81 | 83 | 83 | 85 | 86 | 85 | 78 | 73 | 67 | 64 | 61 | 59 | 58 | 56 | 55 | 56 | 57 | 60 | 65 | 69 | 74 | 76 | 78 | 81 |
| April 29 - May 5 | 7 | 81 | 82 | 82 | 84 | 85 | 81 | 74 | 68 | 63 | 60 | 58 | 57 | 54 | 52 | 53 | 54 | 56 | 58 | 62 | 67 | 70 | 74 | 77 | 79 |
| May 6 - 12 | 7 | 80 | 82 | 84 | 86 | 86 | 80 | 73 | 67 | 61 | 57 | 53 | 50 | 48 | 47 | 48 | 47 | 50 | 53 | 56 | 64 | 69 | 72 | 75 | 78 |
| May 13 - 19 | 7 | 82 | 84 | 85 | 86 | 85 | 79 | 74 | 69 | 64 | 60 | 57 | 54 | 53 | 53 | 52 | 53 | 55 | 56 | 60 | 66 | 71 | 75 | 79 | 81 |
| May 20 - 26 | 7 | 83 | 85 | 86 | 87 | 86 | 82 | 75 | 70 | 66 | 62 | 60 | 57 | 56 | 56 | 56 | 55 | 56 | 57 | 61 | 67 | 74 | 78 | 80 | 83 |
| May 27 - June 2 | 7 | 83 | 85 | 86 | 87 | 84 | 77 | 71 | 66 | 62 | 59 | 54 | 52 | 53 | 52 | 51 | 52 | 52 | 54 | 58 | 65 | 71 | 75 | 78 | 81 |
| June 3 - June 9 | 7 | 86 | 87 | 88 | 88 | 85 | 78 | 71 | 66 | 61 | 57 | 53 | 53 | 51 | 50 | 51 | 52 | 53 | 57 | 59 | 65 | 74 | 79 | 83 | 85 |
| June 10 - 16 | 7 | 82 | 84 | 86 | 87 | 85 | 80 | 77 | 72 | 68 | 65 | 63 | 62 | 62 | 60 | 58 | 60 | 60 | 62 | 64 | 68 | 73 | 77 | 80 | 81 |
| June 17 - 23 | 7 | 85 | 86 | 87 | 88 | 86 | 82 | 76 | 73 | 69 | 66 | 63 | 61 | 59 | 59 | 58 | 58 | 60 | 62 | 64 | 68 | 73 | 77 | 80 | 83 |
| June 24 - 30 | 7 | 85 | 85 | 86 | 88 | 87 | 81 | 74 | 69 | 65 | 62 | 60 | 58 | 56 | 57 | 55 | 59 | 59 | 59 | 62 | 67 | 73 | 78 | 80 | 83 |
| July 1 - 7 | 7 | 81 | 82 | 83 | 83 | 83 | 81 | 77 | 73 | 71 | 69 | 66 | 64 | 64 | 63 | 63 | 63 | 64 | 64 | 67 | 71 | 74 | 77 | 79 | 80 |
| July 8 - 14 | 7 | 84 | 86 | 87 | 88 | 87 | 84 | 79 | 74 | 69 | 66 | 65 | 62 | 61 | 60 | 59 | 61 | 61 | 63 | 66 | 71 | 76 | 79 | 82 | 83 |
| July 15 - 21 | 7 | 88 | 88 | 89 | 90 | 89 | 84 | 77 | 72 | 68 | 65 | 63 | 61 | 59 | 58 | 57 | 56 | 58 | 60 | 63 | 69 | 75 | 79 | 82 | 84 |
| July 22 - 28 | 7 | 87 | 89 | 90 | 91 | 91 | 85 | 78 | 71 | 66 | 62 | 60 | 58 | 58 | 57 | 57 | 57 | 58 | 60 | 64 | 70 | 76 | 81 | 84 | 86 |
| July 29 - Aug 4 | 7 | 87 | 88 | 89 | 90 | 91 | 88 | 82 | 75 | 71 | 69 | 66 | 65 | 63 | 63 | 62 | 61 | 62 | 64 | 67 | 73 | 78 | 82 | 83 | 85 |
| Aug 5 - 11 | 7 | 80 | 83 | 83 | 85 | 85 | 82 | 76 | 70 | 65 | 61 | 57 | 54 | 52 | 51 | 52 | 53 | 56 | 58 | 62 | 69 | 74 | 75 | 77 | 80 |

**Day light**

|  | | Hours | | | | | | | | | | | | | | | | | | | | | | | |
| --- | --- | --- | --- | --- | --- | --- | --- | --- | --- | --- | --- | --- | --- | --- | --- | --- | --- | --- | --- | --- | --- | --- | --- | --- | --- |
| Dates* | Number of the days | 0000 | 0100 | 0200 | 0300 | 0400 | 0500 | 0600 | 0700 | 0800 | 0900 | 1000 | 1100 | 1200 | 1300 | 1400 | 1500 | 1600 | 1700 | 1800 | 1900 | 2000 | 2100 | 2200 | 2300 |
| April 22 - 28 | 7 | 0 | 0 | 0 | 0 | 0 | 0 | 400 | 400 | 400 | 400 | 400 | 400 | 400 | 400 | 400 | 400 | 400 | 400 | 400 | 400 | 400 | 0 | 0 | 0 |
| April 29 - May 5 | 7 | 0 | 0 | 0 | 0 | 0 | 400 | 400 | 400 | 400 | 400 | 400 | 400 | 400 | 400 | 400 | 400 | 400 | 400 | 400 | 400 | 400 | 0 | 0 | 0 |
| May 6 - 12 | 7 | 0 | 0 | 0 | 0 | 0 | 400 | 400 | 400 | 400 | 400 | 400 | 400 | 400 | 400 | 400 | 400 | 400 | 400 | 400 | 400 | 400 | 0 | 0 | 0 |
| May 13 - 19 | 7 | 0 | 0 | 0 | 0 | 0 | 400 | 400 | 400 | 400 | 400 | 400 | 400 | 400 | 400 | 400 | 400 | 400 | 400 | 400 | 400 | 400 | 0 | 0 | 0 |
| May 20 - 26 | 7 | 0 | 0 | 0 | 0 | 0 | 400 | 400 | 400 | 400 | 400 | 400 | 400 | 400 | 400 | 400 | 400 | 400 | 400 | 400 | 400 | 400 | 400 | 0 | 0 |
| May 27 - June 2 | 7 | 0 | 0 | 0 | 0 | 0 | 400 | 400 | 400 | 400 | 400 | 400 | 400 | 400 | 400 | 400 | 400 | 400 | 400 | 400 | 400 | 400 | 400 | 0 | 0 |
| June 3 - June 9 | 7 | 0 | 0 | 0 | 0 | 0 | 400 | 400 | 400 | 400 | 400 | 400 | 400 | 400 | 400 | 400 | 400 | 400 | 400 | 400 | 400 | 400 | 400 | 0 | 0 |
| June 10 - 16 | 7 | 0 | 0 | 0 | 0 | 400 | 400 | 400 | 400 | 400 | 400 | 400 | 400 | 400 | 400 | 400 | 400 | 400 | 400 | 400 | 400 | 400 | 400 | 0 | 0 |
| June 17 - 23 | 7 | 0 | 0 | 0 | 0 | 400 | 400 | 400 | 400 | 400 | 400 | 400 | 400 | 400 | 400 | 400 | 400 | 400 | 400 | 400 | 400 | 400 | 400 | 0 | 0 |
| June 24 - 30 | 7 | 0 | 0 | 0 | 0 | 400 | 400 | 400 | 400 | 400 | 400 | 400 | 400 | 400 | 400 | 400 | 400 | 400 | 400 | 400 | 400 | 400 | 400 | 0 | 0 |
| July 1 - 7 | 7 | 0 | 0 | 0 | 0 | 0 | 400 | 400 | 400 | 400 | 400 | 400 | 400 | 400 | 400 | 400 | 400 | 400 | 400 | 400 | 400 | 400 | 400 | 0 | 0 |
| July 8 - 14 | 7 | 0 | 0 | 0 | 0 | 0 | 400 | 400 | 400 | 400 | 400 | 400 | 400 | 400 | 400 | 400 | 400 | 400 | 400 | 400 | 400 | 400 | 400 | 0 | 0 |
| July 15 - 21 | 7 | 0 | 0 | 0 | 0 | 0 | 400 | 400 | 400 | 400 | 400 | 400 | 400 | 400 | 400 | 400 | 400 | 400 | 400 | 400 | 400 | 400 | 400 | 0 | 0 |
| July 22 - 28 | 7 | 0 | 0 | 0 | 0 | 0 | 400 | 400 | 400 | 400 | 400 | 400 | 400 | 400 | 400 | 400 | 400 | 400 | 400 | 400 | 400 | 400 | 400 | 0 | 0 |
| July 29 - Aug 4 | 7 | 0 | 0 | 0 | 0 | 0 | 400 | 400 | 400 | 400 | 400 | 400 | 400 | 400 | 400 | 400 | 400 | 400 | 400 | 400 | 400 | 400 | 0 | 0 | 0 |
| Aug 5 - 11 | 7 | 0 | 0 | 0 | 0 | 0 | 400 | 400 | 400 | 400 | 400 | 400 | 400 | 400 | 400 | 400 | 400 | 400 | 400 | 400 | 400 | 400 | 0 | 0 | 0 |

**Table S2**. Climate data used in this study for control and drought treatments

*Average climate data of every 7 days during the wheat growing period (April 22nd -August 11th) over the past 5 years (2016–2020) in Malmö, Sweden used in this study.

**Temperature (℃)**

|  | Hours | | | | | | | | | | | | | | | | | | | | | | | |
| --- | --- | --- | --- | --- | --- | --- | --- | --- | --- | --- | --- | --- | --- | --- | --- | --- | --- | --- | --- | --- | --- | --- | --- | --- |
| Dates* | 0000 | 0100 | 0200 | 0300 | 0400 | 0500 | 0600 | 0700 | 0800 | 0900 | 1000 | 1100 | 1200 | 1300 | 1400 | 1500 | 1600 | 1700 | 1800 | 1900 | 2000 | 2100 | 2200 | 2300 |
| April 22 - 28 | 6 | 6 | 6 | 6 | 6 | 7 | 8 | 9 | 10 | 11 | 12 | 12 | 12 | 13 | 12 | 12 | 12 | 11 | 9 | 9 | 8 | 8 | 7 | 7 |
| April 29 - May 5 | 6 | 6 | 6 | 6 | 6 | 7 | 9 | 10 | 10 | 11 | 12 | 12 | 13 | 13 | 13 | 13 | 12 | 11 | 10 | 9 | 8 | 7 | 7 | 6 |
| May 6 - 12 | 8 | 7 | 7 | 7 | 8 | 9 | 11 | 12 | 13 | 14 | 15 | 16 | 16 | 16 | 16 | 16 | 15 | 14 | 13 | 11 | 10 | 9 | 9 | 8 |
| May 13 - 19 | 9 | 9 | 8 | 8 | 9 | 10 | 12 | 13 | 14 | 15 | 16 | 16 | 17 | 17 | 17 | 17 | 16 | 15 | 15 | 13 | 12 | 11 | 10 | 10 |
| May 20 - 26 | 11 | 11 | 11 | 10 | 11 | 13 | 15 | 16 | 17 | 17 | 18 | 19 | 19 | 19 | 19 | 19 | 18 | 18 | 17 | 15 | 14 | 13 | 12 | 12 |
| May 27 - June 2 | 13 | 12 | 12 | 12 | 13 | 15 | 16 | 17 | 18 | 19 | 20 | 21 | 21 | 21 | 21 | 21 | 20 | 20 | 18 | 17 | 15 | 14 | 14 | 13 |
| June 3 - June 9 | 13 | 12 | 12 | 12 | 13 | 15 | 16 | 17 | 19 | 19 | 20 | 20 | 21 | 21 | 21 | 20 | 20 | 19 | 18 | 17 | 15 | 14 | 14 | 13 |
| June 10 - 16 | 14 | 13 | 13 | 13 | 14 | 15 | 16 | 17 | 18 | 19 | 19 | 19 | 20 | 20 | 20 | 20 | 19 | 18 | 18 | 17 | 16 | 15 | 15 | 14 |
| June 17 - 23 | 15 | 14 | 14 | 14 | 15 | 16 | 17 | 18 | 19 | 19 | 20 | 20 | 21 | 21 | 21 | 21 | 20 | 20 | 19 | 18 | 17 | 16 | 16 | 15 |
| June 24 - 30 | 15 | 15 | 14 | 14 | 16 | 17 | 18 | 19 | 20 | 21 | 21 | 22 | 22 | 22 | 23 | 22 | 22 | 21 | 20 | 19 | 18 | 16 | 16 | 15 |
| July 1 - 7 | 14 | 14 | 14 | 14 | 14 | 15 | 16 | 16 | 17 | 17 | 18 | 19 | 19 | 19 | 19 | 19 | 18 | 18 | 18 | 17 | 16 | 15 | 15 | 15 |
| July 8 - 14 | 15 | 14 | 14 | 13 | 14 | 15 | 16 | 17 | 18 | 19 | 19 | 20 | 20 | 20 | 20 | 20 | 20 | 19 | 19 | 17 | 16 | 16 | 15 | 15 |
| July 15 - 21 | 15 | 15 | 14 | 14 | 15 | 16 | 18 | 19 | 19 | 20 | 20 | 21 | 21 | 21 | 21 | 21 | 21 | 20 | 20 | 18 | 17 | 16 | 16 | 15 |
| July 22 - 28 | 16 | 16 | 15 | 15 | 16 | 18 | 19 | 20 | 21 | 22 | 23 | 23 | 23 | 24 | 24 | 23 | 23 | 22 | 21 | 20 | 19 | 18 | 17 | 17 |
| July 29 - Aug 4 | 16 | 16 | 15 | 15 | 16 | 17 | 18 | 19 | 20 | 21 | 21 | 22 | 22 | 22 | 22 | 22 | 22 | 21 | 20 | 19 | 18 | 17 | 17 | 16 |
| Aug 5 - 11 | 17 | 16 | 16 | 16 | 16 | 17 | 18 | 20 | 21 | 21 | 22 | 23 | 23 | 23 | 23 | 22 | 22 | 21 | 20 | 19 | 18 | 18 | 17 | 17 |

**Relative Humidity (%)**

|  | Hours | | | | | | | | | | | | | | | | | | | | | | | |
| --- | --- | --- | --- | --- | --- | --- | --- | --- | --- | --- | --- | --- | --- | --- | --- | --- | --- | --- | --- | --- | --- | --- | --- | --- |
| Dates* | 0000 | 0100 | 0200 | 0300 | 0400 | 0500 | 0600 | 0700 | 0800 | 0900 | 1000 | 1100 | 1200 | 1300 | 1400 | 1500 | 1600 | 1700 | 1800 | 1900 | 2000 | 2100 | 2200 | 2300 |
| April 22 - 28 | 81 | 83 | 83 | 85 | 86 | 85 | 78 | 73 | 67 | 64 | 61 | 59 | 58 | 56 | 55 | 56 | 57 | 60 | 65 | 69 | 74 | 76 | 78 | 81 |
| April 29 - May 5 | 81 | 82 | 82 | 84 | 85 | 81 | 74 | 68 | 63 | 60 | 58 | 57 | 54 | 52 | 53 | 54 | 56 | 58 | 62 | 67 | 70 | 74 | 77 | 79 |
| May 6 - 12 | 80 | 82 | 84 | 86 | 86 | 80 | 73 | 67 | 61 | 57 | 53 | 50 | 48 | 47 | 48 | 47 | 50 | 53 | 56 | 64 | 69 | 72 | 75 | 78 |
| May 13 - 19 | 82 | 84 | 85 | 86 | 85 | 79 | 74 | 69 | 64 | 60 | 57 | 54 | 53 | 53 | 52 | 53 | 55 | 56 | 60 | 66 | 71 | 75 | 79 | 81 |
| May 20 - 26 | 83 | 85 | 86 | 87 | 86 | 82 | 75 | 70 | 66 | 62 | 60 | 57 | 56 | 56 | 56 | 55 | 56 | 57 | 61 | 67 | 74 | 78 | 80 | 83 |
| May 27 - June 2 | 83 | 85 | 86 | 87 | 84 | 77 | 71 | 66 | 62 | 59 | 54 | 52 | 53 | 52 | 51 | 52 | 52 | 54 | 58 | 65 | 71 | 75 | 78 | 81 |
| June 3 - June 9 | 86 | 87 | 88 | 88 | 85 | 78 | 71 | 66 | 61 | 57 | 53 | 53 | 51 | 50 | 51 | 52 | 53 | 57 | 59 | 65 | 74 | 79 | 83 | 85 |
| June 10 - 16 | 82 | 84 | 86 | 87 | 85 | 80 | 77 | 72 | 68 | 65 | 63 | 62 | 62 | 60 | 58 | 60 | 60 | 62 | 64 | 68 | 73 | 77 | 80 | 81 |
| June 17 - 23 | 85 | 86 | 87 | 88 | 86 | 82 | 76 | 73 | 69 | 66 | 63 | 61 | 59 | 59 | 58 | 58 | 60 | 62 | 64 | 68 | 73 | 77 | 80 | 83 |
| June 24 - 30 | 85 | 85 | 86 | 88 | 87 | 81 | 74 | 69 | 65 | 62 | 60 | 58 | 56 | 57 | 55 | 59 | 59 | 59 | 62 | 67 | 73 | 78 | 80 | 83 |
| July 1 - 7 | 81 | 82 | 83 | 83 | 83 | 81 | 77 | 73 | 71 | 69 | 66 | 64 | 64 | 63 | 63 | 63 | 64 | 64 | 67 | 71 | 74 | 77 | 79 | 80 |
| July 8 - 14 | 84 | 86 | 87 | 88 | 87 | 84 | 79 | 74 | 69 | 66 | 65 | 62 | 61 | 60 | 59 | 61 | 61 | 63 | 66 | 71 | 76 | 79 | 82 | 83 |
| July 15 - 21 | 88 | 88 | 89 | 90 | 89 | 84 | 77 | 72 | 68 | 65 | 63 | 61 | 59 | 58 | 57 | 56 | 58 | 60 | 63 | 69 | 75 | 79 | 82 | 84 |
| July 22 - 28 | 87 | 89 | 90 | 91 | 91 | 85 | 78 | 71 | 66 | 62 | 60 | 58 | 58 | 57 | 57 | 57 | 58 | 60 | 64 | 70 | 76 | 81 | 84 | 86 |
| July 29 - Aug 4 | 87 | 88 | 89 | 90 | 91 | 88 | 82 | 75 | 71 | 69 | 66 | 65 | 63 | 63 | 62 | 61 | 62 | 64 | 67 | 73 | 78 | 82 | 83 | 85 |
| Aug 5 - 11 | 80 | 83 | 83 | 85 | 85 | 82 | 76 | 70 | 65 | 61 | 57 | 54 | 52 | 51 | 52 | 53 | 56 | 58 | 62 | 69 | 74 | 75 | 77 | 80 |

**Day light**

|  | Hours | | | | | | | | | | | | | | | | | | | | | | | |
| --- | --- | --- | --- | --- | --- | --- | --- | --- | --- | --- | --- | --- | --- | --- | --- | --- | --- | --- | --- | --- | --- | --- | --- | --- |
| Dates/hours* | 0000 | 0100 | 0200 | 0300 | 0400 | 0500 | 0600 | 0700 | 0800 | 0900 | 1000 | 1100 | 1200 | 1300 | 1400 | 1500 | 1600 | 1700 | 1800 | 1900 | 2000 | 2100 | 2200 | 2300 |
| April 22 - 28 | 0 | 0 | 0 | 0 | 0 | 0 | 400 | 400 | 400 | 400 | 400 | 400 | 400 | 400 | 400 | 400 | 400 | 400 | 400 | 400 | 400 | 0 | 0 | 0 |
| April 29 - May 5 | 0 | 0 | 0 | 0 | 0 | 400 | 400 | 400 | 400 | 400 | 400 | 400 | 400 | 400 | 400 | 400 | 400 | 400 | 400 | 400 | 400 | 0 | 0 | 0 |
| May 6 - 12 | 0 | 0 | 0 | 0 | 0 | 400 | 400 | 400 | 400 | 400 | 400 | 400 | 400 | 400 | 400 | 400 | 400 | 400 | 400 | 400 | 400 | 0 | 0 | 0 |
| May 13 - 19 | 0 | 0 | 0 | 0 | 0 | 400 | 400 | 400 | 400 | 400 | 400 | 400 | 400 | 400 | 400 | 400 | 400 | 400 | 400 | 400 | 400 | 0 | 0 | 0 |
| May 20 - 26 | 0 | 0 | 0 | 0 | 0 | 400 | 400 | 400 | 400 | 400 | 400 | 400 | 400 | 400 | 400 | 400 | 400 | 400 | 400 | 400 | 400 | 400 | 0 | 0 |
| May 27 - June 2 | 0 | 0 | 0 | 0 | 0 | 400 | 400 | 400 | 400 | 400 | 400 | 400 | 400 | 400 | 400 | 400 | 400 | 400 | 400 | 400 | 400 | 400 | 0 | 0 |
| June 3 - June 9 | 0 | 0 | 0 | 0 | 0 | 400 | 400 | 400 | 400 | 400 | 400 | 400 | 400 | 400 | 400 | 400 | 400 | 400 | 400 | 400 | 400 | 400 | 0 | 0 |
| June 10 - 16 | 0 | 0 | 0 | 0 | 400 | 400 | 400 | 400 | 400 | 400 | 400 | 400 | 400 | 400 | 400 | 400 | 400 | 400 | 400 | 400 | 400 | 400 | 0 | 0 |
| June 17 - 23 | 0 | 0 | 0 | 0 | 400 | 400 | 400 | 400 | 400 | 400 | 400 | 400 | 400 | 400 | 400 | 400 | 400 | 400 | 400 | 400 | 400 | 400 | 0 | 0 |
| June 24 - 30 | 0 | 0 | 0 | 0 | 400 | 400 | 400 | 400 | 400 | 400 | 400 | 400 | 400 | 400 | 400 | 400 | 400 | 400 | 400 | 400 | 400 | 400 | 0 | 0 |
| July 1 - 7 | 0 | 0 | 0 | 0 | 0 | 400 | 400 | 400 | 400 | 400 | 400 | 400 | 400 | 400 | 400 | 400 | 400 | 400 | 400 | 400 | 400 | 400 | 0 | 0 |
| July 8 - 14 | 0 | 0 | 0 | 0 | 0 | 400 | 400 | 400 | 400 | 400 | 400 | 400 | 400 | 400 | 400 | 400 | 400 | 400 | 400 | 400 | 400 | 400 | 0 | 0 |
| July 15 - 21 | 0 | 0 | 0 | 0 | 0 | 400 | 400 | 400 | 400 | 400 | 400 | 400 | 400 | 400 | 400 | 400 | 400 | 400 | 400 | 400 | 400 | 400 | 0 | 0 |
| July 22 - 28 | 0 | 0 | 0 | 0 | 0 | 400 | 400 | 400 | 400 | 400 | 400 | 400 | 400 | 400 | 400 | 400 | 400 | 400 | 400 | 400 | 400 | 400 | 0 | 0 |
| July 29 - Aug 4 | 0 | 0 | 0 | 0 | 0 | 400 | 400 | 400 | 400 | 400 | 400 | 400 | 400 | 400 | 400 | 400 | 400 | 400 | 400 | 400 | 400 | 0 | 0 | 0 |
| Aug 5 - 11 | 0 | 0 | 0 | 0 | 0 | 400 | 400 | 400 | 400 | 400 | 400 | 400 | 400 | 400 | 400 | 400 | 400 | 400 | 400 | 400 | 400 | 0 | 0 | 0 |

**Table S3**. Effect of genotype, stress environment (treatment) and their interaction on the gluten protein parameters analyzed by SE-HPLC. LPP and SPP designate large and small polymeric proteins (extractable and unextractable (s), respectively), LMP and SMP (extractable and unextractable (s), respectively.)

| Factors | Df | LPP  10^14^ | SPP  10^14^ | LMP  10^15^ | SMP  10^13^ | LPPs  10^14^ | SPPs  10^14^ | LMPs  10^14^ | SMPs  10^12^ |
| --- | --- | --- | --- | --- | --- | --- | --- | --- | --- |
| Genotype (G) | 7 | 1.10*** | 1.46*** | 2.63*** | 4.16*** | 0.82*** | 0.86*** | 0.37** | 0.65 |
| Treatment (T) | 3 | 1.12*** | 2.94*** | 7.12*** | 2.82*** | 3.60*** | 2.87*** | 1.40*** | 4.99*** |
| G x T | 21 | 0.46 | 0.69 | 1.52 | 3.43* | 1.11** | 1.07* | 0.49 | 0.65 |
| Residues | 96 | 2.11 | 2.96 | 5.42 | 8.83 | 2.37 | 2.70 | 1.39 | 7.21 |

**Table S4.** Post hoc Tukey’s test showing the effect of treatments on the gluten protein parameters studied by SE-HPLC. LPP and SPP designate large and small polymeric proteins (extractable and unextractable (s), respectively), LMP and SMP (extractable and unextractable (s), respectively).

| Treatment | LPP  10^6^ | SPP  10^7^ | LMP  10^8^ | SMP  10^6^ | LPPs  10^6^ | SPPs  10^7^ | LMPs  10^6^ | SMPs  10^6^ |
| --- | --- | --- | --- | --- | --- | --- | --- | --- |
| Control | 6.19 b | 1.06 bc | 3.70 b | 7.62 bc | 3.30 c | 4.34 c | 3.88 bc | 0.81 b |
| Drought | 5.16 c | 0.98 c | 3.60 b | 7.61 bc | 3.61 bc | 4.72 bc | 3.78 c | 0.65 b |
| Heat | 5.22 c | 1.1 b | 4.07 b | 8.03 ab | 4.45 b | 5.51 b | 4.61 b | 0.81 b |
| Combined | 7.47 a | 1.38 a | 5.46 a | 8.40 a | 7.53 a | 8.18 a | 6.39 a | 1.15 a |
